# The deubiquitinase USP9X and E3 ligase WWP1 orchestrate IGF2BP2 ubiquitination homeostasis to drive TNBC progression and cisplatin sensitivity

**DOI:** 10.1038/s41419-025-08038-5

**Published:** 2025-10-06

**Authors:** Tian Xia, Jianyi Zhao, Zhengyu Zhang, Weilin Lu, Yuxin Wang, Xinrui Dong, Mingyi Sang, Linjie Ju, Xu Zhang, Jifu Wei, Qiang Ding

**Affiliations:** 1 Jiangsu Breast Disease Center & Department of General Surgery, The First Affiliated Hospital with Nanjing Medical University, Nanjing, China; 2 Department of Pharmacy, Jiangsu Cancer Hospital & Jiangsu Institute of Cancer Research & The Affiliated Cancer Hospital of Nanjing Medical University, Nanjing, China

**Keywords:** Breast cancer, Ubiquitylation

## Abstract

The functional impact of post-translational modifications (PTMs) on many N6-methyladenosine (m6A) regulatory proteins remains unclear. Our previous study demonstrated that the m6A reader IGF2BP2 drives triple-negative breast cancer (TNBC) progression through epigenetic regulation. Here, we found that IGF2BP2 ubiquitination homeostasis was dynamically regulated by the opposing actions of USP9X (deubiquitinase) and WWP1 (E3 ligase). We further identified USP9X as a cisplatin-binding protein, whose inactivation upon cisplatin treatment shifts this balance toward WWP1-mediated IGF2BP2 degradation in TNBC. This suppressed IGF2BP2-mediated stabilization and translation of m6A-modified MYC/CDK6 mRNAs, thereby inhibiting TNBC progression. Notably, combined USP9X inhibitor WP1130 and low-dose cisplatin showed synergistic therapeutic efficacy against TNBC in both in vivo and in vitro models. Overall, our findings established that the USP9X/WWP1 axis maintained IGF2BP2 ubiquitination homeostasis to regulate m6A-dependent oncogenic functions in TNBC. Crucially, cisplatin uniquely disrupts this balance through USP9X binding, impairing IGF2BP2’s m6A recognition capacity and revealing a novel UPS-mediated drug response mechanism specific to TNBC treatment.

## Introduction

Triple-negative breast cancer (TNBC) is a subtype of breast cancer with the worst prognosis and highly prone to recurrence and metastasis [1]. Due to its negative presentation of estrogen receptor (ER), progesterone receptor (PR) and human epidermal growth factor receptor 2 (HER2), it lacks an effective therapeutic target and is not sensitive to endocrine therapy, HER2-targeted therapy [2]. While immune checkpoint inhibitors and Poly ADP-Ribose Polymerase Inhibitor (PARP) inhibitors have expanded treatment options beyond conventional chemotherapy for TNBC, challenges such as drug resistance and variable efficacy across molecular subtypes persist [3, 4]. Therefore, investigating novel TNBC targets and optimizing combination regimens may lead to improved clinical outcomes.

N6-methyladenosine (m6A), the most abundant mRNA modification in eukaryotes, is dynamically regulated by m6A writers, erasers, and readers, and plays critical roles in cancer pathogenesis [5]. We previously demonstrated that Insulin-like growth factor 2 mRNA-binding protein (IGF2BP) 2, an m6A reader overexpressed in TNBC, accelerated tumor progression by regulating m6A-dependent cyclin-dependent kinase (CDK) 6 translation and cell proliferation [6]. However, the development of highly selective inhibitors is extremely challenging due to the fact that the K homology (KH) structural domains, which play important roles such as recognizing m6A sites and maintaining the stability of targeted genes and translational output functions, are present in a wide range of RNA-binding proteins [7, 8]. In addition, due to the structural similarity between m6A readers, their different functions in different normal or tumor cells and their simultaneous involvement in multiple biological processes, especially in TNBC show a strong heterogeneity. It is difficult to achieve the expected effect of low-selective IGF2BP2 inhibitors [9]. Therefore, directly targeting of post-translational modifications (PTMs) processes of proteins, by precisely targeting the biological processes of m6A-modified proteins in the species of malignant tumor progression by directly affecting their stability or enzymatic activity, has great potential for exploration [10].

Ubiquitination is the most common and important type of PTMs, which governs targeted proteins via the E1-E2-E3 cascade, modulating their stability, function, localization, and interactions [11]. Deubiquitinating enzymes (DUBs) reverse ubiquitination by removing ubiquitin from targeted proteins, regulating key processes like protein stability and signaling. This dynamic reversibility, balanced by coordinated E3 ligase and DUB activity, is crucial for proteostasis [12]. Recently about the functional impact of ubiquitination on m6A modification-related proteins has been gradually emphasized. Wang et al. found that ubiquitin specific peptidase (USP) 47 prevented the ubiquitination of the m6A reader protein YTH N6-methyladenosine RNA binding protein (YTHDF) 1, which attenuated the translational output of c-MYC proto-oncogene (MYC) and coordinately maintained the metabolic homeostasis of tumor-infiltrating Tregs [13]; Xu et al. revealed that F-box and WD repeat domain-containing 7‌‌ (FBW7) was able to ubiquitinate the degradation of the m6A reader YTHDF2 and affected the apoptotic process in ovarian cancer [14]; Zeng et al. found that Methyltransferase-like (METTL) 3 promoted the process of m6A modification in tumor cells by competitively binding to the m6A writer METTL14 with STIP1 homology and U-Box containing protein 1 (STUB1) in order to prevent it from being degraded [15]; Chang et al. identified USP36 as the a DUB that protected the m6A eraser alkylation repair homolog 5 (ALKBH5) and promoted malignant progression of glioblastoma [16]. However, the regulatory mechanisms of IGF2BP2 by the ubiquitin system, particularly its role in TNBC malignancy, remain poorly understood and require further investigation.

Here in this study, we demonstrated that in TNBC, the m6A reader IGF2BP2 is dynamically regulated by USP 9X-linked (USP9X)-mediated deubiquitination and WW domain containing E3 ubiquitin protein ligase 1 (WWP1)-mediated ubiquitination, maintaining its critical functions in m6A recognition, target mRNA stabilization, and translational control. Guan et al. verified that targeted silencing of USP9X could increase the cisplatin sensitivity of TNBC [17]. Wei et al. analyzed clinical data and found that cisplatin treatment efficacy and prognosis of esophageal squamous cell carcinoma patients with high expression of USP9X were significantly worse [18]. In this study, we interestingly identified USP9X as a direct cisplatin-binding protein by pull-down assays with biotinylated cisplatin probes coupled with mass spectrometry. The binding of cisplatin to USP9X disrupts its deubiquitylation activity toward IGF2BP2, shifting the equilibrium toward WWP1-mediated ubiquitination and proteasomal degradation in TNBC. This loss of IGF2BP2 stability impairs its m6A reader function, suppressing oncogenic mRNA stabilization and translation, thereby inhibiting TNBC tumor growth. Our findings delineate a non-traditional cytotoxic mechanism of cisplatin that targets the ubiquitin-proteasome system, fundamentally distinct from its classical DNA crosslinking activity, and establish the USP9X/WWP1-IGF2BP2 axis as a novel therapeutic targeting strategy for TNBC.

## Results

### USP9X is a deubiquitinating enzyme that potentially affects IGF2BP2 protein stability in TNBC

**Fig. 1:**
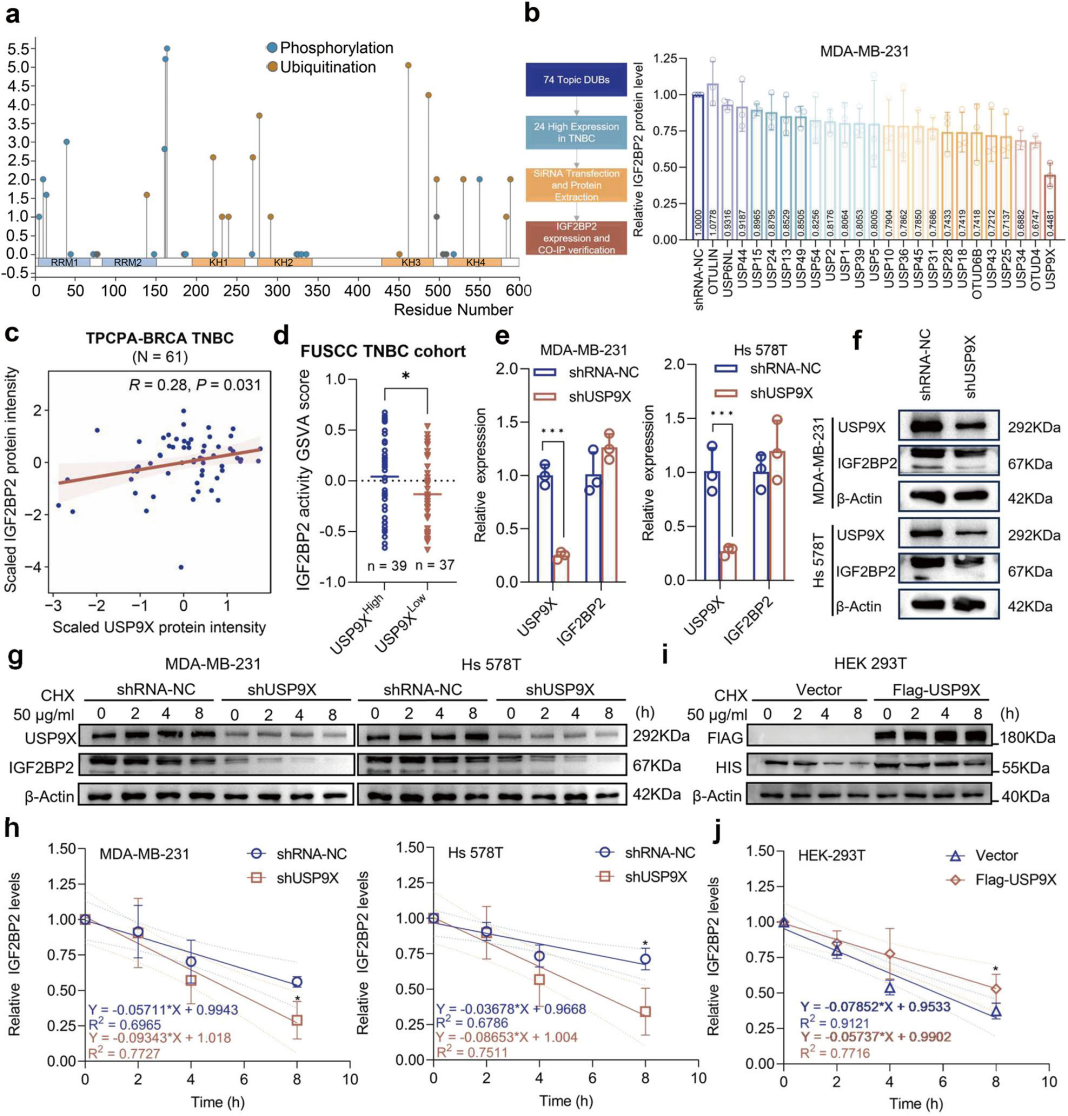
USP9X affects the protein expression level and stability of IGF2BP2. **a** Demonstration of the PTMs locus of IGF2BP2 by the PhosphoSitePlus database. **b** Flowchart showing the route to construct the siRNA screen; IGF2BP2 expression was assessed by Western blot upon DUB knockdown, with densitometric analysis of band gray values. **c** A positive correlation was observed between IGF2BP2 and USP9X protein levels in Pan-Cancer Proteome Atlas (TPCPA)-BRCA TNBC (*R* = 0.28, *P* = 0.031), with missing values imputed using a k-nearest neighbors- based algorithm. **d** IGF2BP2 activity is higher in USP9X-high tumors than in USP9X-low tumors in the FUSCC TNBC cohort. **e**, **f** Expression levels of USP9X in MDA-MB-231 and Hs 578T cells transfected with knockdown USP9X (shUSP9X) and blank control (shRNA-NC) were detected by qRT-PCR and Western blot. **g**, **h** Western blot analysis of IGF2BP2 protein levels in MDA-MB-231 and Hs 578T cells stably transfected with shUSP9X or control shRNA (shRNA-NC) following treatment with 50 μg/ml cycloheximide (CHX) for the indicated time points (0, 2, 4, 6, 8 h). Quantification of grayscale values from all experimental groups was performed to determine the protein degradation half-life (t₁/₂) of IGF2BP2. **i**, **j** HEK 293T cells were co-transfected with HIS-IGF2BP2 and FLAG-USP9X, followed by treatment with 50 μg/ml CHX to inhibit protein synthesis. Cells were harvested at the indicated time points (0, 2, 4, 6, 8 h), and protein levels were analyzed by Western blot using anti-HIS (for IGF2BP2) and anti-FLAG (for USP9X) antibodies. Quantification of grayscale values from all experimental groups was performed to determine the protein degradation half-life (t₁/₂) of HIS-IGF2BP2. Error bars indicate mean (*n* = 3) ± standard deviation. *P < 0.05, **P < 0.01, ***P < 0.001.

We explored the major types of PTMs present on the IGF2BP2 protein by PhosphoSitePlus and noted that the key KH structural domains of IGF2BP2 as an m6A reader protein was regulated by numerous ubiquitination modifications (Fig. 1a). Considering the essential role of DUBs as part of the ubiquitination regulatory system in the maintenance of protein stability and essential functions, we analyzed the expression levels of 74 topical DUBs in TNBC using bcGenExMiner on a case-by-case basis (Supplementary Table 1), screened out 24 highly-expressed DUBs and designed their siRNAs and transfected them into MDA-MB-231 cells. Western blot analysis by gray value presentation revealed that the protein level of IGF2BP2 was significantly reduced after knockdown of USP9X (Figs. 1b and S1a). Based on GSE96058, GSE81538 databases, it was further clarified that IGF2BP2 and USP9X showed high expression in TNBC (Fig. S1b, c). We then analyzed protein expression data from TNBC samples in the Pan-Cancer Proteome Atlas (TPCPA) database and found a positive correlation between IGF2BP2 and USP9X protein levels but not in mRNA levels (Figs. 1c and S1d). And further studies based on the FUSCC TNBC cohort showed that IGF2BP2 activity was significantly higher in the USP9X high-expression group than in the USP9X low-expression group (*p* = 0.04125) (Fig. 1d). In addition, using the Kaplan–Meier Plotter database, we stratified TNBC samples into three cohorts: all TNBC cases, USP9X^High^ subgroup (expression above median), and USP9X^Low^ subgroup (expression below median). Consistent with our previous findings, elevated IGF2BP2 expression strongly correlated with poor prognosis in the overall TNBC cohort [6]. Notably, this association was particularly pronounced in USP9X^High^ samples, whereas no significant correlation was observed in USP9X^Low^ samples (Fig. S1e–g). These results indicate that USP9X may primarily exert its oncogenic effects through modulation of IGF2BP2 protein levels. Four USP9X siRNAs with different sequences were transfected individually and a virally stable transcriptional knockdown cell line for USP9X was constructed based on si-2, which has the best silencing efficiency (Fig. S1b). qRT-PCR and western blot verified the transfection efficiency and confirmed that USP9X affected IGF2BP2 expression only at protein level and had no significant effect on its mRNA level (Fig. 1e, f). Upon further blocking of the nascent protein synthesis using CHX, we found that reduced USP9X expression in the TNBC cells resulted in the destabilization of the IGF2BP2 protein (Fig. 1g, h); whereas, after forced overexpression of USP9X in HEK 293T cells, a significant increase in the stability of the HIS-labled IGF2BP2 protein was observed (Fig. 1i, j).

### USP9X interacts with IGF2BP2

**Fig. 2:**
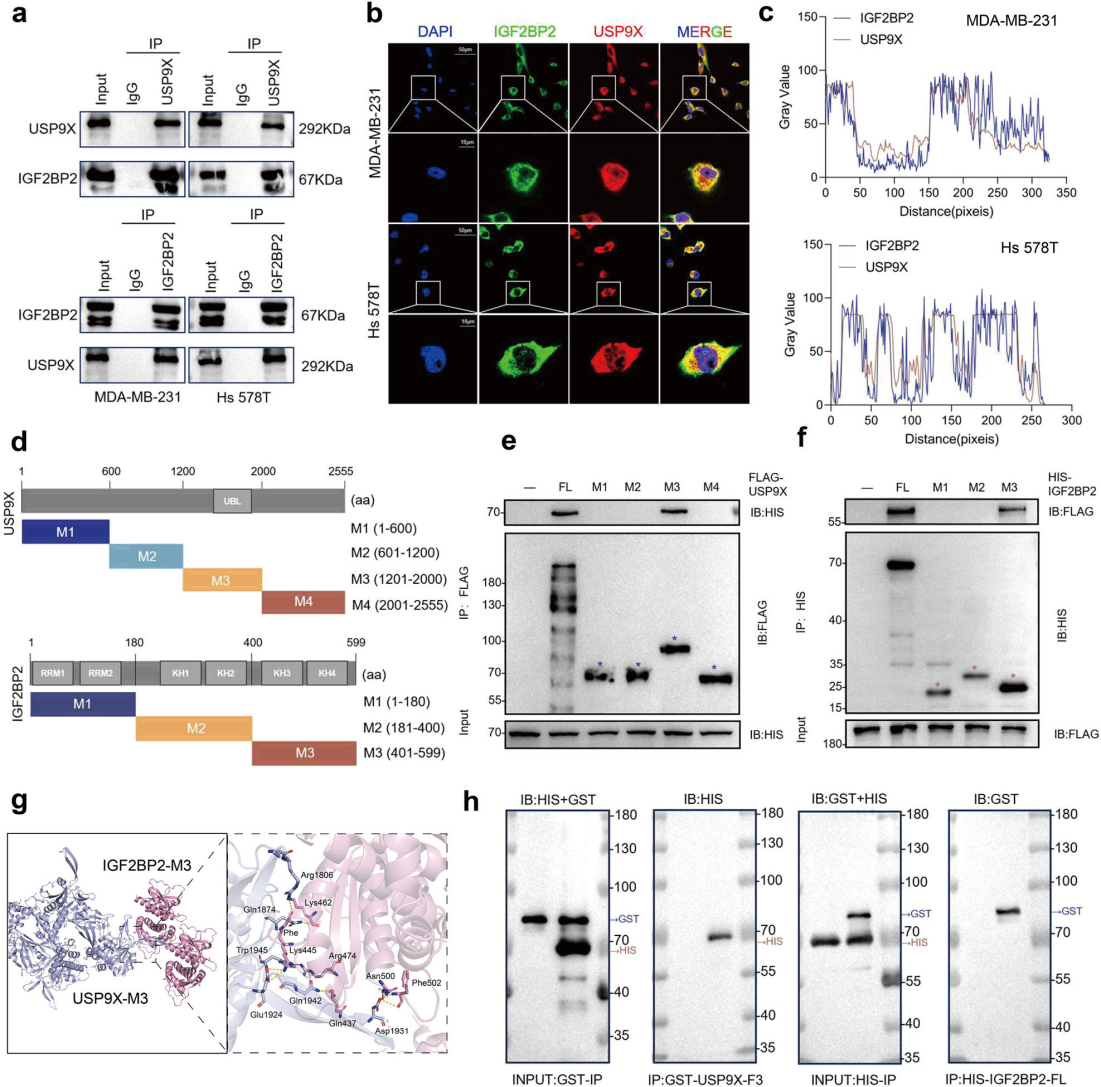
USP9X interacts with IGF2BP2. **a** Cell lysates of MDA-MB-231 and Hs 578T were pulled down by IP using antibodies against USP9X and IGF2BP2, followed by Western blot detection. **b**, **c** Confocal images showing co-localization of USP9X (red) and IGF2BP2 (green) in MDA-MB-231 and Hs 578T cells, and nuclei were restained with DAPI (blue), followed by quantitative analysis of fluorescence intensity. **d** Domain organization schematics of FLAG-USP9X, HIS-IGF2BP2 and their engineered truncation constructs, depicting functional domains and truncation boundaries. **e** HEK 293T cells were co-transfected with HIS-IGF2BP2 and FLAG-tagged USP9X full-length or truncated mutants. Cell lysates were analyzed by IP with FLAG beads and then detected by Western blot with antibodies against HIS and FLAG. **f** HEK 293T cells were cotransfected with FLAG-USP9X and HIS-tagged full-length or truncated mutants of IGF2BP2. Cell lysates were analyzed by IP with HIS beads and then detected by Western blot with antibodies against FLAG and HIS. **g** HDock (http://hdock.phys.hust.edu.cn/) analysis showing details of the interaction between USP9X-M3 and IGF2BP2-M3, the protein models were retrieved and downloaded at UniProt (https://www.uniprot.org/). **h** Purified GST-USP9X-M3 and HIS-IGF2BP2 were incubated in binding buffer at 4 °C for 2 h, Complexes were pulled down with HIS/GST magnetic beads, Input (10%) and bound fractions were analyzed by Western blot.

We next attempted to determine whether IGF2BP2 interacted directly with USP9X. By Co-IP assay, it could be determined that the physical association between endogenous IGF2BP2 and USP9X proteins was validated in TNBC cells (Fig. 2a). Confocal images further clarified that IGF2BP2 (green) and USP9X (red) were co-localized in the cytoplasm of TNBC cells (Fig. 2b), and quantitative analysis of fluorescence intensities confirmed this finding (Fig. 2c). To specify the minimum imperative region for its binding, we designed HIS-tagged IGF2BP2 and FLAG-tagged USP9X and their truncated mutants based on the protein length and their unique structural domains (Fig. 2d). IP assays confirmed that the M3 region (401-599), where the KH3-4 structural domain of IGF2BP2 was located, and the M3 (1201-2000), where the UBL structural domain of USP9X was located, were the main regions mediating their interactions (Fig. 2e, f). The X-RAY structures 6ROL (IGF2BP2-M3) and 5WCH (USP9X-M3) provided by UNIPORT, which contain the above regions, were used for further molecular docking simulations to show clearer details of protein interactions (Fig. 2g). Further, we purified the HIS-tagged IGF2BP2 full length protein and performed in vitro protein interaction pull-down experiments with the purified protein of the GST-tagged USP9X M3 segment (HY-P701431, MCE, NJ, USA) to further clarify its interconnections (Fig. 2h).

### USP9X regulates K48-linked deubiquitination of IGF2BP2 at residues K487 and K583

**Fig. 3:**
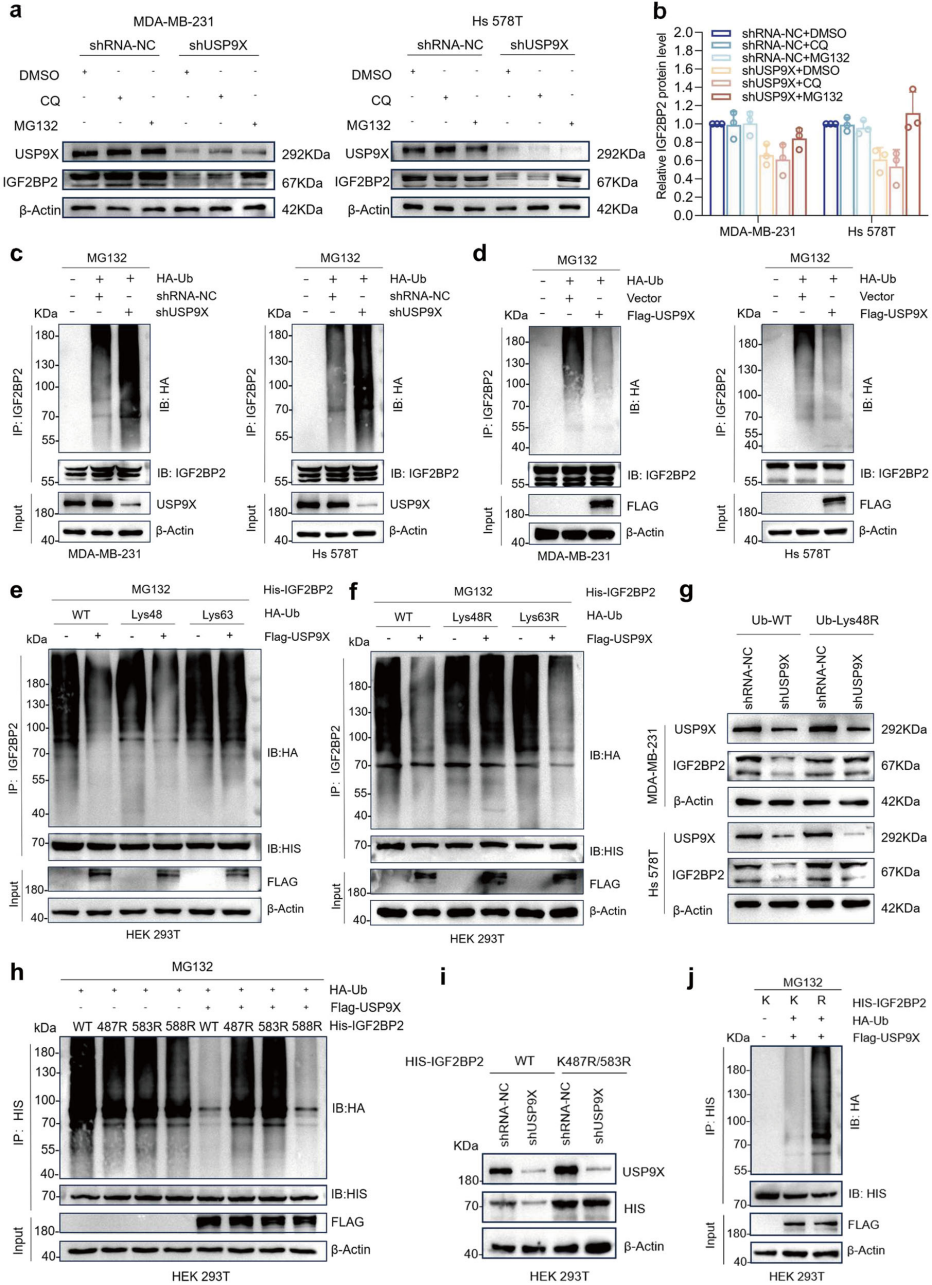
USP9X deubiquitinates IGF2BP2. **a**, **b** Total proteins were extracted from control and USP9X-knockdown MDA-MB-231 and Hs 578T cells treated with DMSO, MG132, or CQ for 8 h. IGF2BP2 protein levels were analyzed by Western blot, and band intensities were quantified for statistical comparison. Error bars indicate mean (*n* = 3) ± standard deviation. **c**, **d** MDA-MB-231 and Hs 578T cells were co-transfected with HA-Ub along with either control siRNA, USP9X-targeting siRNA (siUSP9X), or FLAG-USP9X plasmid. Following MG-132 (20 μM, 6 h) treatment to stabilize ubiquitinated proteins, IGF2BP2 was immunoprecipitated and its ubiquitination status was analyzed by Western blot using anti-HA antibody. **e**, **f** HEK 293T cells were cotransfected using HIS-tagged IGF2BP2 with HA-Ub, Lys48 only, Lys63 only, Lys48R, Lys63R plasmids, respectively, and after treatment with 20 μM MG-132 for 6 h. HIS pull-down assays were performed and ubiquitination patterns were analyzed by immunoblotting with anti-HA for ubiquitin conjugates and anti-HIS for IGF2BP2 loading control. **g** HA-Ub wild-type or Ub-Lys48R was transfected in MDA-MB-231 and Hs 578T cells in control or knockdown USP9X groups. After 72 h, total protein was extracted and IGF2BP2 levels were analyzed by Western blot using anti-IGF2BP2 antibody, with USP9X detection serving as knockdown verification. **h** HEK293T cells were co-transfected with FLAG-USP9X, HA-Ub, and either wild-type HIS-IGF2BP2 or its lysine-to-arginine (K-R) mutants. Following MG-132 treatment (20 μM, 6 h) to stabilize ubiquitinated proteins, HIS-tagged IGF2BP2 was immunoprecipitated and ubiquitination patterns were analyzed by immunoblotting with anti-HA (ubiquitin), anti-HIS (IGF2BP2), and anti-FLAG (USP9X). **i** HEK 293T cells were cultured for 72 h after co-transfection with empty or siUSP9X using wild-type Ub, HIS-IGF2BP2 or HIS-IGF2BP2487/583R plasmids, and total proteins were collected and subjected to Western blot. Quantification of HIS-IGF2BP2 protein levels normalized to β-Actin. **j** HIS-tagged IGF2BP2-WT or HIS-IGF2BP2-487/583R was used to co-transfect with HA-Ub and FLAG-USP9X, and ubiquitin ligation of IGF2BP2 was analyzed after collection of total proteins after treatment with 20 μM MG-132 for 6 h and IP pull-down using HIS antibody.

To further clarify the specific role played by USP9X in protecting IGF2BP2 from degradation, we used lysosomal inhibitor (Chloroquine diphosphate, CQ) and protease inhibitor (MG132) for pretreatment, and the results showed that MG132 reversed the IGF2BP2 protein level due to the knockdown of USP9X downregulation, suggesting that USP9X protects IGF2BP2 from degradation by the proteasomal pathway rather than the lysosomal pathway mainly through its deubiquitination function (Fig. 3a, b). Further knockdown of USP9X revealed a significant increase in ubiquitination modification of IGF2BP2 in TNBC cells, whereas its ubiquitination level was significantly downregulated after forced expression of USP9X (Fig. 3c, d). To date, Lys48- or Lys63-linked ubiquitin chains are the two predominant linkage types in the ubiquitination-proteasome pathway, and deubiquitination assays in the HEK 293T cell line demonstrated that USP9X significantly reduced K48-linked polyubiquitination of IGF2BP2 (Fig. 3e). In contrast, forced expression of USP9X did not effectively remove K48-linked polyubiquitination after transfection of K48R and K63R (only Lys48 or Lys63 mutated to Arg) mutants according to the same experimental method (Fig. 3f). The reduction in IGF2BP2 expression triggered by USP9X knockdown was reversed after further expression of K48R in USP9X-knockdown TNBC cells, demonstrating that polyubiquitination of the Lys48 linkage was essential for USP9X-regulated IGF2BP2 degradation (Fig. 3g). We predicted IGF2BP2 general and E3-specific lysine ubiquitination sites based on GPS-Uber and PhosphoSitePlus and designed three mutants (IGF2BP-K487R, K583R, K588R) for the ubiquitination assay based on scoring values (Fig. S3a). Upon co-expression of USP9X and mutants in HEK 293T cells, USP9X did not effectively remove the ubiquitination modifications at the K487 and K583 sites (Fig. 3h). In addition, HIS-IGF2BP2 expression due to USP9X knockdown was reverted after simultaneous mutation of K487/K583R (Fig. 3i), while overexpression of USP9X was not effective in removing its polyubiquitination modification (Fig. 3j). In summary, USP9X mediates its protein stability by removing K48-linked polyubiquitination at residues K487 and K583 of IGF2BP2.

### WWP1 interacts with IGF2BP2 and maintains its ubiquitin homeostasis equilibrium with USP9X

**Fig. 4:**
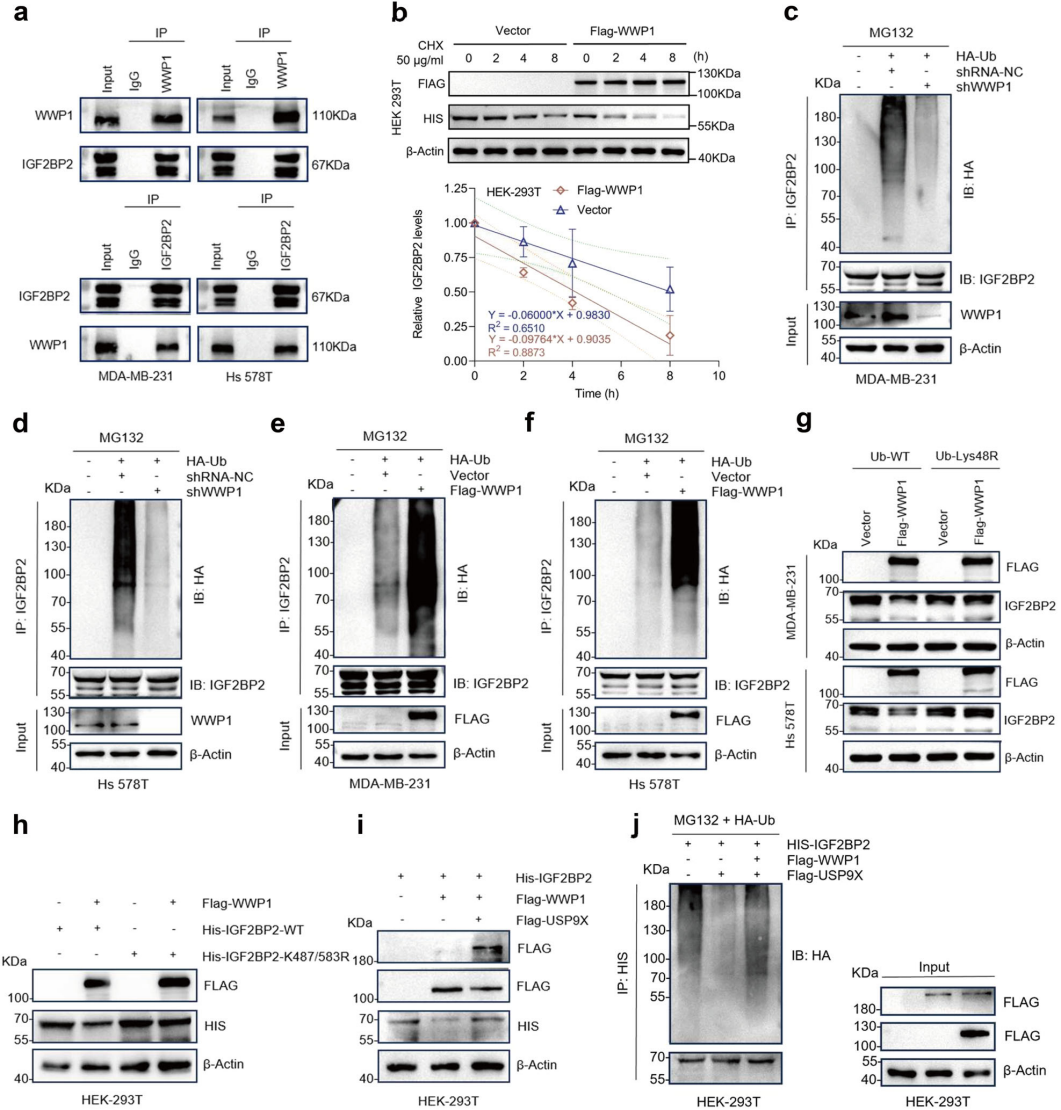
WWP1 interacts with IGF2BP2 and maintains IGF2BP2 ubiquitin homeostasis equilibrium with USP9X. **a** Cell lysates of MDA-MB-231 and Hs 578T were pulled down by IP using antibodies against WWP1 and IGF2BP2, followed by Western blot detection. **b** HEK293T cells were co-transfected with HIS-tagged IGF2BP2 and FLAG-tagged WWP1, treated with CHX (50 μg/ml) and total proteins were collected at the corresponding time and then detected by Western blot, and the gray values were calculated for statistical analysis. Error bars indicate mean (*n* = 3) ± standard deviation. **c**–**f** MDA-MB-231 and Hs 578T were co-transfected with HA-Ub using the indicated controls, siWWP1 or FLAG-tagged WWP1, and after treatment with 20 μM MG-132 for 6 h. IGF2BP2 was immunoprecipitated and its ubiquitination status was analyzed by immunoblotting with anti-HA antibody (for ubiquitin conjugates) and anti-IGF2BP2 antibody (for protein input control). **g** HA-Ub wild-type or Ub-Lys48R was transfected in MDA-MB-231 and Hs 578T cells transfected with empty vector or FLAG-tagged WWP1 groups, and the total proteome was collected after 72 h and detected by Western blot using IGF2BP2 and FLAG antibodies. **h** HEK 293T cells were cultured with wild-type Ub, HIS-IGF2BP2 or HIS-IGF2BP2-487/583R plasmids co-transfected with null or FLAG-WWP1 for 72 h. Total proteins were collected and subjected to Western blot. **i** HEK293T cells were co-transfected with HIS-IGF2BP2 and empty vector (control), FLAG-WWP1, FLAG-USP9X, or both FLAG-WWP1 and FLAG-USP9X, along with wild-type ubiquitin. After 72 h, total protein was extracted and IGF2BP2 expression levels were analyzed by immunoblotting with anti-HIS antibody, while ectopic WWP1 and USP9X expression was verified using anti-FLAG antibody. **j** HEK 293T cells were cotransfected with HA-Ub, HIS-IGF2BP2 with empty or FLAG-USP9X or FLAG-USP9X and FLAGWWP1 and treated with 20 μM MG-132 for 6 h. After treatment with HIS antibody, the cells were subjected to IP pull-down and the ubiquitination levels of HIS-IGF2BP2 were ultimately assessed by Western blot analysis.

The homeostasis of the proteasome-ubiquitination system is mainly maintained by the interactions between E3 ligases and DUBs [12]. As it was found the existence of mutual antagonism between USP9X and the E3 ligase WWP1 in MDA-MB-231 cells, we sought to explore the potential of this combination in coordinating the ubiquitin homeostasis of targeted proteins [19]. Co-IP assay further verified that there was indeed an interaction between IGF2BP2 and WWP1 (Fig. 4a), while overexpression of WWP1 significantly accelerated the degradation of HIS-IGF2BP2 after treatment of HEK 293T cells with CHX (Fig. 4b). After validating and transfecting siWWP1 with the best knockdown efficiency (Fig. S4a), we transfected Lys48-only plasmids in both MDA-MB-231 and Hs 578T cells. Knockdown of WWP1 significantly reduced the ubiquitination of IGF2BP2 compared to control (Fig. 4c, d). In contrast, the ubiquitination level of IGF2BP2 was significantly upregulated after overexpression of WWP1 (Fig. 4e, f), and transfection of K48R reversed the degradation of IGF2BP2 caused by WWP1 overexpression (Fig. 4g). In addition, the expression of HIS-IGF2BP2 caused by overexpression of WWP1 was reverted and its ubiquitination level rebounded after simultaneous mutation of K487/583R (Figs. 4h and S4b). The above results confirmed the important role of WWP1 for IGF2BP2 in the process of K48-linked polyubiquitination. To further validate the antagonistic effect of USP9X on WWP1, we overexpressed USP9X in HEK 293T cells co-transfected with HIS-IGF2BP2 and FLAG-WWP1, and the results confirmed that the expression level of HIS-IGF2BP2 was reverted by overexpression of USP9X (Fig. 4i). In contrast, overexpression of WWP1 in a system co-transfected with HIS-IGF2BP2 and FLAG-USP9X disrupted the level of low ubiquitination modification of IGF2BP2, which was maintained due to USP9X, and ended up with a significant increase (Fig. 4j). Furthermore, WWP1 expression analysis in TNBC based on GSE96058 and GSE81538 databases revealed consistently low levels comparable to non-TNBC controls (Fig. S4c). Survival analysis demonstrated no statistically significant association between WWP1 expression and TNBC prognosis (Fig. S4d). These findings suggest WWP1 likely functions primarily as a synergistic regulator within the USP9X-IGF2BP2 axis rather than as an independent prognostic factor. In summary, WWP1 is an essential E3 ligase in the homeostasis of ubiquitination of IGF2BP2 and maintains this balance together with USP9X.

### Cisplatin disrupts the homeostatic balance of IGF2BP2 in TNBC

**Fig. 5:**
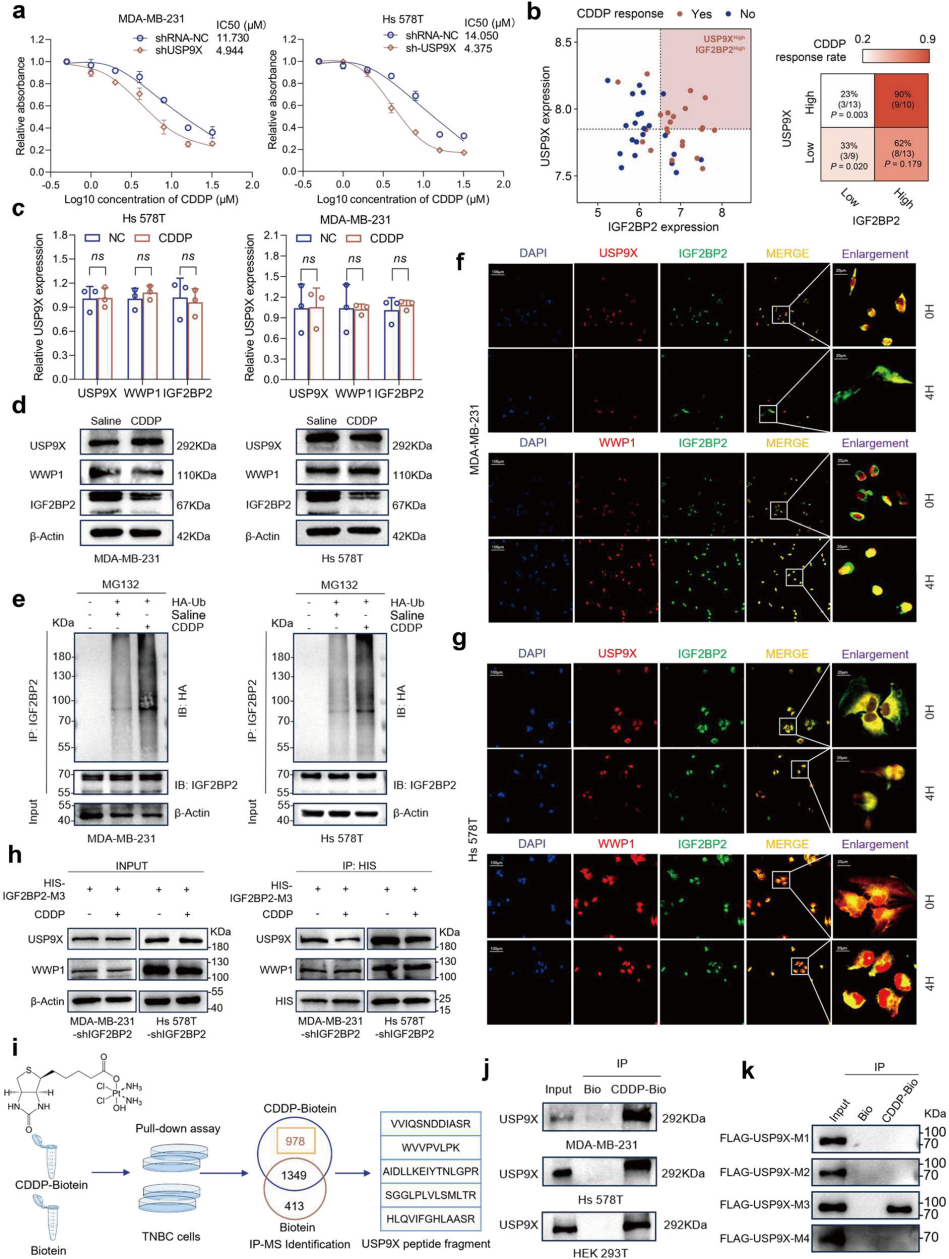
Cisplatin disrupts the homeostatic balance of IGF2BP2 in TNBC. **a** CCK-8 assay was used to examine the IC_50_ value of Cisplatin (CDDP, MCE, HY-17394). **b** Scatter plot of IGF2BP2 and USP9X expression in individual patients, with color indicating cisplatin response status. Dotted lines represent median values of IGF2BP2 and USP9X expression, used to define high and low expression subgroups. Fisher ’s exact test was used to compare the IGF2BP2High/USP9XHigh group against each of the other three subgroups. The response rate was significantly higher than that in the IGF2BP2Low/USP9XHigh group (*P* = 0.003) and the IGF2BP2Low/USP9XLow group (*P* = 0.020), and showed a non-significant trend toward higher response compared to the IGF2BP2High/USP9XLow group (*P* = 0.179). Heatmap colors indicate response rates. **c**, **d**. Expression levels of USP9X, WWP1 and IGF2BP2 in MDA-MB-231 and Hs 578T cells from saline control (NC) and cisplatin drug-treated (CDDP) groups were detected by qRT-PCR and Western blot. **e** HA-Ub was transfected in NC and CDDP group cells, respectively, and IP pull-down was performed using IGF2BP2 antibody and detected by Western blot. **f**, **g** Confocal images showing co-localization of USP9X or WWP1 (red) and IGF2BP2 (green) in MDA-MB-231 and Hs 578T cells after 4 h treatment with saline or CDDP for 4 h, with nuclei were restained with DAPI (blue). **h** His-tagged IGF2BP2-M3 was overexpressed in IGF2BP2-knockdown MDA-MB-231 and Hs 578T cells treated with cisplatin or saline control. Total proteins were IP using anti-HIS antibody, followed by Western Blot assays with antibodies against USP9X, WWP1, and HIS-tag. **i** Biotin-labeled cisplatin probes were synthesized for pull-down assay and the enriched proteins were used for mass spectrometry identification. The identification results comprehensively listed all unique peptide sequences specific to USP9X. **j** Cell lysates from MDA-MB-231, Hs 578T, and HEK293T cells were incubated with biotin-labeled cisplatin probes or biotin-only controls coupled to streptavidin magnetic beads. After pull-down and denaturation, captured proteins were analyzed by Western blot with USP9X antibody to specifically detect cisplatin-USP9X interactions. **k** Four FLAG-tagged USP9X truncation mutants were purified as recombinant proteins for in vitro binding assays. These proteins were incubated with streptavidin magnetic beads conjugated to either biotin-cisplatin or biotin alone. Bound proteins were eluted and analyzed by immunoblotting with anti-FLAG antibody. Error bars indicate mean (*n* = 3) ± standard deviation. *P < 0.05, **P < 0.01, ***P < 0.001.

Previous studies have suggested that USP9X is considered a potential cisplatin sensitizing gene [17, 18], and the drug sensitivity to cisplatin was significantly increased in TNBC cells after knockdown of USP9X (Fig. 5a). We then assessed the association between USP9X/IGF2BP2 co-expression and cisplatin sensitivity in two independent cohorts of breast cancer patients undergoing cisplatin-based neoadjuvant chemotherapy [20, 21]. Results showed that samples exhibiting concurrent high expression of USP9X and IGF2BP2 demonstrated significantly enhanced therapeutic response to cisplatin (Fig. 5b), further suggesting that USP9X-mediated cisplatin sensitization is primarily through its targeted regulation of IGF2BP2. Cells treated with cisplatin for 6 h were collected and subjected to qRT-PCR and Western blot. It was found that only the protein level of IGF2BP2 was downregulated but the mRNA level was not affected, while neither USP9X nor WWP1 was changed, suggesting that the change in the protein level of IGF2BP2 may be triggered by the destabilization of its protein by cisplatin (Fig. 5c, d). We further treated TNBC cells transfected with Ub plasmid using both MG132 and cisplatin, and treatment with cisplatin more significantly increased the level of ubiquitination of IGF2BP2 as compared to control (Fig. 5e). Confocal images also visualized that after cisplatin treatment, in addition to some changes in cell morphology, the degree of co-localization of IGF2BP2 with USP9X decreased, whereas the degree of co-localization with WWP1 was significantly enhanced (Figs. 5f, g, and S5a, b). Subsequently, upon overexpression of HIS-tagged IGF2BP2-M3 in virally stabilized TNBC cells with IGF2BP2 knockdown, compared to the saline-treated control group, cisplatin treatment significantly reduced USP9X enrichment bound to the IGF2BP2-M3 segment in Co-IP assays. Notably, WWP1 binding remained unchanged (Fig. 5h). These results indicate that cisplatin specifically disrupts USP9X-IGF2BP2-M3 interaction. The above results suggested that cisplatin might destabilize the USP9X/WWP1-IGF2BP2 axis by directly binding to a particular protein, thereby breaking the USP9X/WWP1-IGF2BP2 axis. To further advance this conjecture, we designed and synthesized biotin-labeled cisplatin probes for pull-down experiments and silver staining experiments, and we were surprised to find USP9X identified in the subsequent MS identification results (Figs. 5i, and S6a and b). Western blot results further confirmed that USP9X could indeed be pulled down and enriched by biotin-labeled cisplatin (Fig. 5j). We then performed in vitro binding assays using recombinant truncated USP9X mutants to evaluate interactions with cisplatin. The results demonstrated that only the M3 segment directly binds cisplatin (Fig. 5k). Additionally, we indirectly evaluated the deubiquitinating activity of USP9X and E3 ligase activity of WWP1 by monitoring ubiquitination levels of DVL2—a reported substrate co-regulated by both enzymes [19]. Co-IP assays were conducted in HEK 293T cells expressing DVL2 following cisplatin gradient treatment, with subsequent Western blot analysis. Results revealed cisplatin selectively abrogated USP9X deubiquitinase activity while sparing WWP1 E3 ligase function (Fig. S6c, d). Interestingly, the UBL domain essential for USP9X catalytic activity resides within its M3 segment. To conclude, cisplatin destabilizes the ubiquitin equilibrium of IGF2BP2 by directly binding to USP9X, thereby destabilizing the protein of IGF2BP2.

### Cisplatin disrupts the function of IGF2BP2 as an m6A reader

**Fig. 6:**
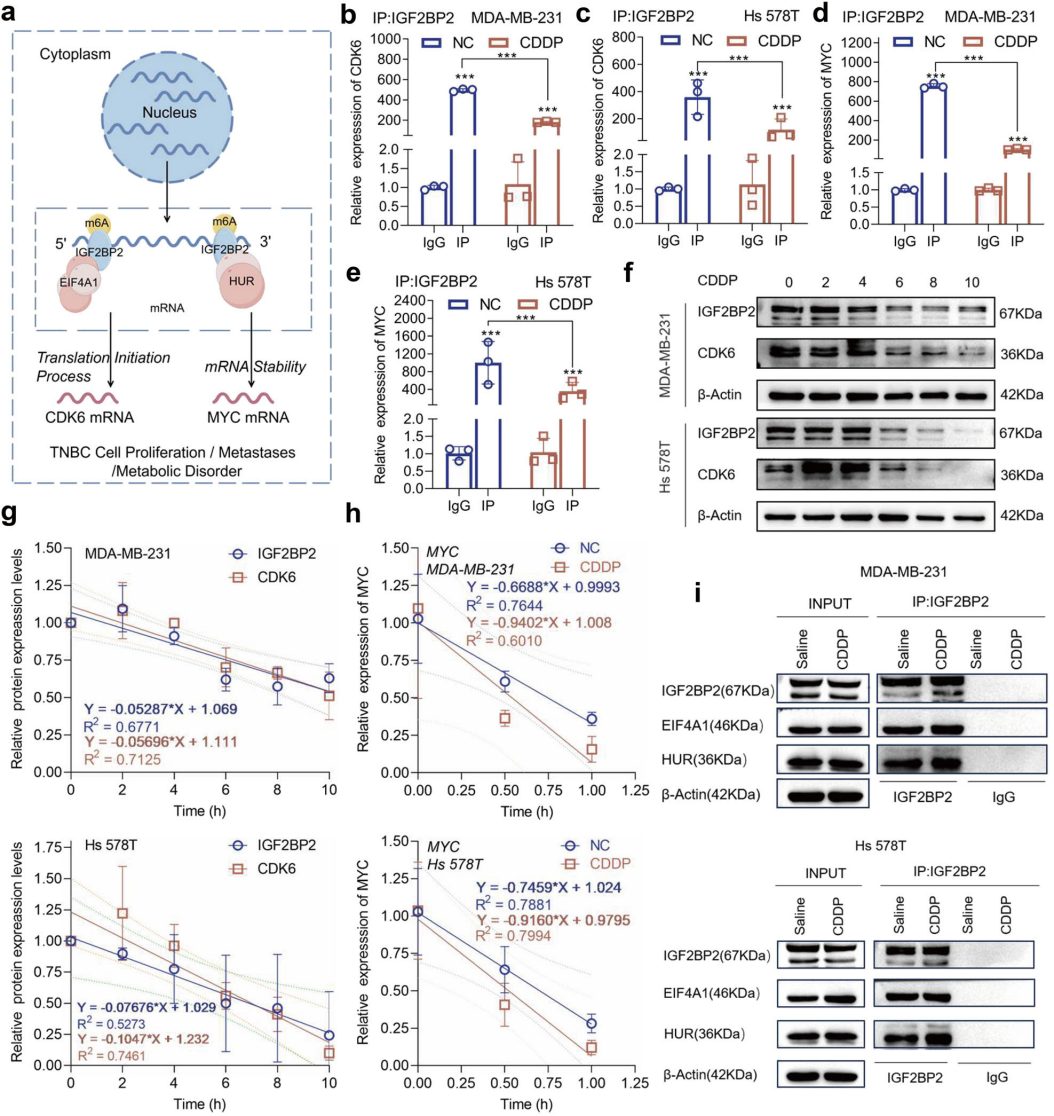
Cisplatin disrupts the function of IGF2BP2 as an m6A reader. **a** Working model of IGF2BP2 targeting MYC and CDK6 mRNAs in an m6A-dependent manner to promote TNBC progression. **b**–**e** qRT-PCR was performed to measure the transcript levels of CDK6 and MYC within IGF2BP2 or IgG immunocomplexes in saline or cisplatin-treated MDA-MB-231 and Hs 578T cell lysates. Relative levels of all genes were normalized with IgG. **f** After treatment with CHX (50 μg/ml), the corresponding total proteins were collected at different time points of cisplatin addition and protein abundance of IGF2BP2 and CDK6 was detected by Western blot. **g** Protein abundance of IGF2BP2 and CDK6 was made to be converted to gray values and analyzed statistically. **h** mRNA half-life (t1/2) was measured in cisplatin-treated vs control cells using actinomycin D (5 μg/mL) chase (0, 0.5, 1 h) followed by qRT-PCR. **i** MDA-MB-231 and Hs 578 T cells were treated with either saline or cisplatin for 4 h. IGF2BP2-containing complexes were immunoprecipitated from cell lysates and analyzed by immunoblotting for interacting partners (HUR and EIF4A1), with IGF2BP2 serving as the immunoprecipitation control. Error bars indicate mean (*n* = 3) ± standard deviation. *P < 0.05, **P < 0.01, ***P < 0.001.

The KH3-4 structural domain of IGF2BP2 is the key structure for its function as an m6A reader [22]. Our previous studies have fully demonstrated that IGF2BP2 promotes the translational output of CDK6 through the recruitment of Eukaryotic Initiation Factor 4A1 (EIF4A1) and thus promotes the rapid proliferation of TNBC [6], relevant studies have also amply demonstrated that IGF2BP2 promotes malignant metastasis and metabolic progression of cancer cells by recruiting Human antigen R (HUR) to target MYC [7, 23] (Fig. 6a). We first tested the ability of cisplatin treatment on the exercise of RNA-binding proteins by IGF2BP2, and the RNA-binding ability of IGF2BP2 for CDK6 and MYC mRNA was significantly decreased after cisplatin treatment compared with the untreated group (Fig. 6b–e). Subsequently, after cisplatin treatment and simultaneous blockade of de novo protein production by CHX, the protein levels of IGF2BP2 and CDK6 maintained a similar trend of decreasing, suggesting that cisplatin treatment affected the translational output of IGF2BP2 to CDK6 (Fig. 6f, g). Changes in the stability of MYC mRNA were detected by adding actinomycin D at two time points, the last 1 h and 0.5 h of cisplatin pretreatment, and the results showed that cisplatin treatment resulted in a dramatic decrease in the stability of MYC (Fig. 6h). Finally, we examined the interaction of cisplatin treatment on IGF2BP2 and the interaction proteins EIF4A1 and HUR, and Western blot results showed that its interconnection with the two proteins was not affected by cisplatin (Fig. 6i). The above experimental results indicated that cisplatin disrupted the function of IGF2BP2 as an m6A reader-binding targeted gene affecting its mRNA stability and translational output, but not its ability to recruit interaction proteins.

### USP9X inhibitors are synergistic with cisplatin treatment in TNBC

**Fig. 7:**
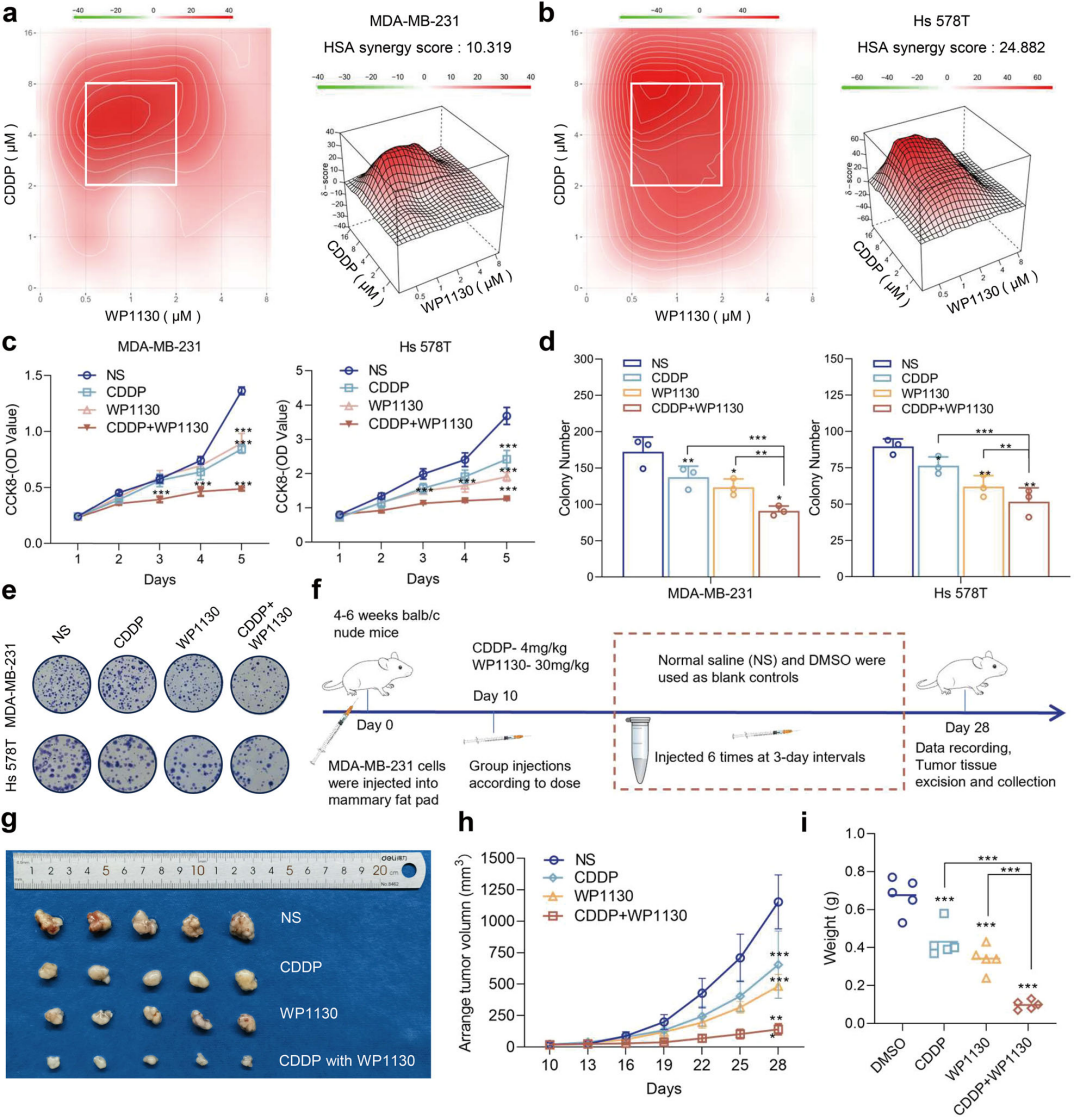
Targeting USP9X increases TNBC’s sensitivity to cisplatin. **a**, **b** Synergy finder 3.0 analyzes and visualizes the results of checkerboard experiments with different concentrations of co-administered WP1130 and cisplatin (CDDP), with drug combinations having the strongest synergistic effects marked by white squares. **c** Growth of MDA-MB-231 and Hs 578T cells after 5 days of treatment with different drugs was measured using the CCK8 assay. **d**, **e**. Cell growth was measured using a colony formation assay over 14 days of different drug treatments and statistically analyzed after counting. **f** Flowchart constructing a nude mouse xenograft tumor model and demonstrating the detailed protocol for drug delivery, parts of the graphic material was obtained from SciDraw (https://scidraw.io/). **g**–**i** The resected tumor mass was photographed and statistically analyzed for volume and mass. Error bars indicate mean (*n* = 3) ± standard deviation. *P < 0.05, **P < 0.01, ***P < 0.001.

Cisplatin may inhibit TNBC progression by binding to USP9X and disrupting its protection against the deubiquitination of IGF2BP2, thereby inhibiting it as an m6A reader, which raises the question of whether the USP9X inhibitor WP1130 and Cisplatin can be used in combination at low doses for larger killing of TNBC cells. Synergy finder 3.0 was used to calculate the drug association index of WP1130 and CDDP in TNBC cells, and the HSA synergy score showed that the two drugs exhibited positive synergistic effects (Figs. 7a, b, and S7a). We next chose the drug combination of WP1130 = 1 μM and cisplatin = 4 μM, which were able to significantly inhibit the proliferation rate and clone formation ability of TNBC cells at relatively low concentrations (Fig. 7c–e). Meanwhile, the combination regimen showed no adverse effects on the proliferative capacity of normal breast epithelial cells MCF-10A (Fig. S7b–d), further confirming the safety and reliability of this therapeutic strategy. We then constructed a nude mouse xenograft tumor model (Fig. 7f). By observing the tumor growth in the blank control group (injected with saline and corn oil), the single WP1130 group, the single cisplatin group, and the WP1130 and cisplatin drug combination group, we obtained results consistent with the in vitro experiments (Fig. 7f–i). In summary, the addition of USP9X inhibitor further weakened the protection of USP9X against deubiquitination of IGF2BP2 and increased the ubiquitination degradation of IGF2BP2 under cisplatin treatment, and thus further inhibited the rapid progression of TNBC.

## Discussion

Though the essential role of the m6A reader protein IGF2BP2 in its promotion of multiple processes such as mRNA translation, splicing, and export has been revealed [24, 25], the factors affecting the function of the m6A reader IGF2BP2 protein are still poorly understood, and the regulation of PTMs to which it is subjected remains unclear. In this study, we revealed that the m6A reader protein IGF2BP2 was synergistically regulated by the DUB USP9X and the E3 ligase WWP1, which affected IGF2BP2 protein stability and function through the balance of this dynamic ubiquitin modification. In addition, we demonstrated for the first time that the cisplatin-binding protein USP9X caused ubiquitination degradation by exposure of the KH3/4 structural domain of IGF2BP2 to WWP1 during cisplatin treatment of TNBC cells, leading to an unexpected positive combined therapeutic effect of the USP9X inhibitor WP1130 with cisplatin in TNBC.

As an important class of m6A reader proteins, the IGF2BP family (IGF2BPs) has been gradually emphasized by researchers as an emerging field of study for the regulation of PTMs to which it is subjected. Harald et al. identified the phosphorylation state of IGF2BP1 as an important factor in the regulation of ribonucleoprotein condensate formation and RNA metabolism [26]; Arash et al. revealed that the state of SIRT1-mediated acetylation of IGF2BP2 had a significant impact on its ability to mediate the process of catalytic transcript degradation [27]; Lu et al. found that lactylation of IGF2BP3 and the loop regulation of its downstream lactate products was an important defense mechanism for antioxidant protection of tumor cells [28]. However, the effects of PTMs on the KH domain - a critical structural element for IGF2BP’s m6A recognition, localization, and binding functions - remain poorly understood. The KH domain contains conserved amino acid residues that form a specific functional structure, with reactive side chains susceptible to various chemical modifications, making this domain particularly prone to PTMs [7, 8, 29]. Our study revealed extensive ubiquitination modifications on IGF2BP2’s KH domain, indicating its structural stability depends on ubiquitination homeostasis. Notably, these modifications did not impair IGF2BP2’s scaffold function in recruiting HUR and EIF4A to sustain targeted mRNA stability and translation. Furthermore, in the FUSCC TNBC cohort, USP9X protein levels correlated with IGF2BP2 activity [30], further suggesting that USP9X-mediated deubiquitination is crucial for maintaining IGF2BP2’s transcriptional control over its targeted genes. Admittedly our exploration of PTMs for IGF2BP2 still had limitations that did not take into account the progression of TNBC in numerous states of existential stress, such as the metabolic dysregulation of TNBC resulting in the production and aberrant accumulation of lactate and glutathione that will inevitably trigger the progression of other protein modifications of IGF2BP2 [31, 32], which we will continue to explore in the follow-up as well.

Our study demonstrated that USP9X deubiquitinates IGF2BP2, while WWP1 counteracts this effect by promoting IGF2BP2 degradation, establishing a bidirectional regulatory mechanism for precise IGF2BP2 homeostasis control. Such DUB-E3 ligase coordination - involving either mutual constraint or coregulation - plays crucial roles in cell signaling, DNA repair, and tumor suppression [12, 33]. As an example, under normal physiological conditions USP7 inhibits E3 ligase mousedouble minute 2 (MDM2) degradation, thereby inhibiting P53 signaling, whereas under DNA damage, USP7 directly deubiquitinates and stabilizes P53 to maintain cellular homeostasis [34, 35]; TNF receptor associated factor 6 (TRAF6) and tumor necrosis factor alpha-Induced protein 3 (TNFAIP3‌‌), on the other hand, affect autophagosome formation by regulating the degree of K63-linked ubiquitination of Beclin 1 [36]. And notably, Casey et al. revealed the details of the synergistic modification of the co-targeted protein disheveled segment polarity protein 2 (DVL2) by USP9X and WWP1 for the first time in MDA-MB-231 cells, and found that the redundant WW domains of WWP1 scaffold interactions with both USP9X and DVL2 through PY motif binding, while WW1/WW3 domains specifically mediated DVL2 engagement. This USP9X/WWP1 axis established a ubiquitin rheostat on DVL2, and in this system, USP9X-mediated deubiquitination is essential for maintaining target protein stability and function. Importantly, WWP1 overexpression significantly impairs DVL2 function specifically under USP9X-deficient conditions, demonstrating this regulatory dependence [19]. Our study also verified the important function of USP9X-WWP1, a ubiquitin rheostat maintaining ubiquitin homeostasis, on the IGF2BP2 protein, providing richer evidence for therapeutic strategies to enrich ubiquitin homeostasis and target m6A-modified ubiquitin homeostasis in the life process of tumor cells.

We identified USP9X as a direct cisplatin-binding protein by protein pull-down mass spectrometry and Western blot validation with a designed biotin-labeled cisplatin probe, suggesting that cisplatin appeared to target tumor cell death not only by interacting with DNA to form a cross-linking complex as traditionally perceived, but may have other unintended roles as well. Xavier et al. found that cisplatin was not densely distributed in chromatin, but rather unevenly distributed in substructures of the nucleus and throughout the cell by high-definition multi-ion beam imaging (HD-MIBI) [37]. Hu et al. found that cisplatin promoted drug resistance in non-small cell lung cancer by directly binding and activation of estrogen receptor beta (ERβ), suggesting that cisplatin was able to bind directly to intracellular proteins, exerting biological effects such as affecting gene transcription that are different from traditional killing mechanisms [38]. Whereas, our experimental results revealed that cisplatin disrupted the ubiquitin homeostasis of IGF2BP2 maintained by USP9X/WWP1, as evidenced by the impairment of the m6A recognition function of IGF2BP2 and a decrease in protein abundance. Furthermore, we demonstrated that cisplatin directly binds to the M3 domain (amino acids 1201–2000) of USP9X, a region containing the UBL domain that is essential for deubiquitinase activity, and specifically inhibits this enzymatic function. Notably, cisplatin treatment does not impair WWP1’s E3 ligase activity. These findings reveal that cisplatin-induced disruption of ubiquitin homeostasis creates permissive conditions for WWP1-mediated ubiquitination and degradation of IGF2BP2. And this effect coincided with the pro-apoptotic effect of cisplatin in synergy, explaining the significant increase in drug sensitivity to cisplatin of TNBCs after knocking down USP9X.

Based on the above proposed possible mechanisms, we evaluated the drug combination effect of USP9X inhibitor WP1130 and cisplatin in TNBC, and found that their combination at their respective low doses could produce a strong killing effect on TNBC. The use and exploration of platinum-containing drugs in the neoadjuvant and postoperative adjuvant treatment of TNBC has gradually entered a white-hot phase [39], and the CALGB 40603 study found that the addition of carboplatin to standard neoadjuvant chemotherapy (paclitaxel + anthracyclines) resulted in a positive increase in the pathologic complete remission (pCR) rate in the carboplatin group [40]; The S1416 study evaluated the efficacy of cisplatin in combination with a PARP inhibitor (Veliparib) in TNBC patients with BRCA mutations and showed that the combination significantly prolonged progression-free survival, while platinum-containing combination regimens for patients with non-BRCA mutations remain to be explored [41]; The TONIC trial also validated that the addition of cisplatin to TNBC chemotherapy regimens may induce a tumor immune microenvironment more conducive to PD-L1 monoclonal antibodies treatment [42]. The DNA damage mechanism of cisplatin tends to make investigators more inclined to carry out therapeutic studies in TNBC patients with BRCA-like status [39], while conventional doses of cisplatin are prone to renal damage, ototoxicity, and susceptibility to drug resistance [43], so our proposed combination of USP9X inhibitors with cisplatin for TNBC showed great potential to be explored in the context of expanding the indications for TNBC patients and alleviating the need for a reduced dose of the combination therapy.

**Fig. 8:**
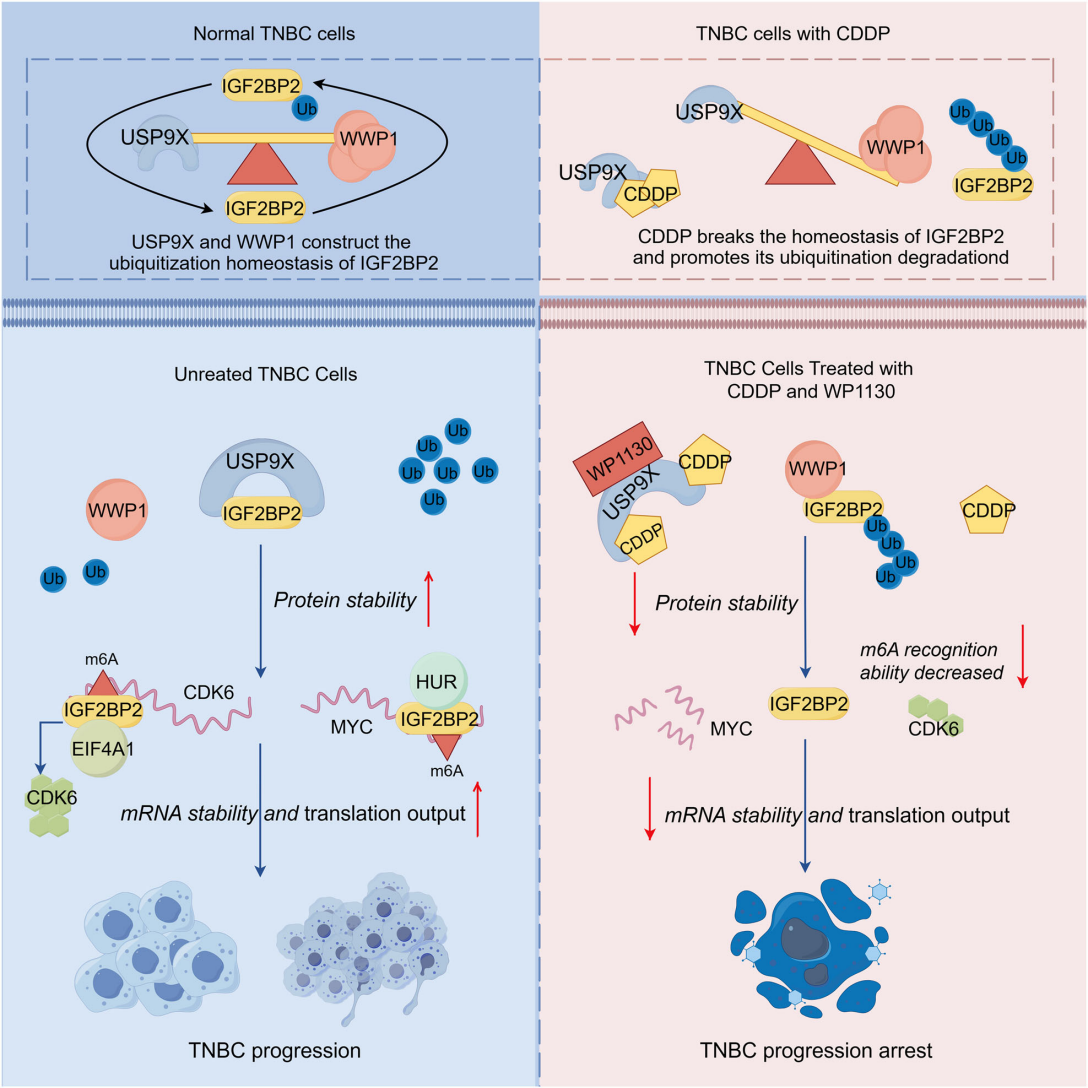
The mechanism chart of this study. Mechanistically, USP9X and WWP1 coordinately control IGF2BP2 stability through ubiquitination modulation. Cisplatin (CDDP) binding to USP9X disrupts this balance. Therapeutically, combining WP1130 (USP9X inhibitor) with cisplatin ablates IGF2BP2’s m6A-binding activity toward MYC and CDK6 mRNAs, providing an effective strategy against TNBC progression.

Here in this study, we demonstrated that USP9X and WWP1 jointly maintain IGF2BP2 ubiquitination homeostasis, with USP9X playing a critical role in preserving IGF2BP2 stability and m6A recognition capacity. Cisplatin disrupts this equilibrium by binding USP9X, shifting the balance toward WWP1-mediated IGF2BP2 degradation and consequent loss of oncogenic mRNA stabilization. The synergistic efficacy of WP1130 with cisplatin underscores the therapeutic promise of co-targeting this regulatory axis, potentially revitalizing platinum-based strategies for TNBC (Fig. 8).

## Experimental section

### Cell culture

MDA-MB-231 (RRID: CVCL_0062), Hs 578T (RRID: CVCL_0332), HEK 293T (RRID: CVCL_0063) and MCF-10A (RRID: CVCL_0598) cells were procured from the ATCC and cultured in Dulbecco Modified Eagle Medium (DMEM) (Wisent, China) or MCF 10A Cell-Specific Culture Medium (Pricella, China) containing 10% fetal bovine serum (FBS) and 1% Penicillin/Streptomycin/Amphotericin B at 37 °C in a 5% CO_2_ atmosphere. Cell line identity was verified through STR authentication, ensuring absence of misidentification and cross-contamination, and cells were used within 10 passages to minimize phenotypic drift.

### Plasmids, siRNA, shRNA and transfection

FlAG-labeled USP9X, HIS-labeled IGF2BP2 and its mutants, HA-labeled ubiquitin, HA-labeled ubiquitin-Lys48 (K48)/Lys63 (K63), FlAG -labeled WWP1 were designed and produced by the Public Protein/Plasmid Library (PPL, Nanjing, China). The siRNAs were designed and synthesized by Sangon Biotech (Shanghai, China). Lentiviral shRNA constructs were obtained from GenePharma (Shanghai, China), specific sequences were based on the siRNA with the best silencing efficiency, siRNA and shRNA sequences used in this study were listed in Supplementary Table 2.

Lentiviral vectors were transfected with the assistance of polybrene (HY-112735, MCE, NJ, USA) and then screened by adding puromycin in a concentration gradient until a final concentration of 3 μg/ml. Transfection of plasmids and siRNAs were performed according to the instructions provided by Lipofectamine^TM^ 3000 Reagent (Invitrogen, Carlsbad, CA, USA), and sample wells were harvested after 48–72 h to verify transfection efficiency.

### RNA isolation and quantitative real time polymerase chain reaction (qRT-PCR)

Total RNA was extracted using the Cellular RNA Rapid Extraction Kit (Goonie, Guangzhou, China) and reverse transcribed using HiScript II Q RT SuperMix for qPCR (+gDNA wiper) (R223-01, Vazyme, Nanjing, China) to obtain cDNA after detection of the concentration. qRT-PCR was performed using AceQ qPCR SYBR Green Master Mix (High Rox Premixed) (Q111-02/03, Vazyme, Nanjing, China) and LightCycler480 II (Roche, Basel, Switzerland). qPCR primer sequences were listed in Supplementary Table 3.

### Western blot analysis, antibodies and reagents

After counting cells and washing them twice with PBS, followed by lysed using RIPA Lysis buffer containing protease and phosphatase Inhibitor Cocktail (HY-K0013, MCE, NJ, USA) and denatured by SDS-PAGE using Omni-Easy™ Instant protein sampling buffer (LT101, Epizyme Biotech, Shanghai, China), separated by SDS-PAGE and transferred to a PVDF membrane (Millipore, MA, USA). The membranes were added to protein-free rapid blocking solution (PS108P, Epizyme Biotech, Shanghai, China) and incubated with primary and secondary antibodies sequentially for detection. The antibodies used were listed in Supplementary Table 4.

### Co-immunoprecipitation (Co-IP)

Cells were fully lysed and collected using Immunoprecipitation (IP) lysis buffer (P0013, Beyotime, Shanghai, China) supplemented with protease and phosphatase Inhibitor Cocktail, and then incubated overnight at 4 °C with the linkage of the targeted antibody protein A/G magnetic beads (HY-K0202, MCE, NJ, USA) with uniform rotation. The enriched proteins were washed on the second day and denatured for Western blot analysis.

### Protein stability assay

The indicated cells were treated with cycloheximide (CHX, HY-12320, MCE, NJ, USA) at a concentration of 50 μg/ml and then lysed and collected according to time points. Protein half-lives were calculated after electrophoresis according to grey value analysis.

### Expression and purification of recombinant HIS or FLAG-tagged proteins

HIS-IGF2BP2, FLAG-USP9X-M1/2/3/4 was purified using the Magneti-Q His-tagged Protein Purification Kit (BK1002, ACE Bio, Nanjing, China). HEK293T cells transfected with plasmid (48–72 h) were lysed with 1% protease/phosphatase inhibitor cocktail and 1 μg/ml nuclease (per 3 × 10⁷ cells). The lysate was incubated with pre-washed Magneti-Q Anti-HIS or FlAG Beads, followed by five 1×PBST washes. Input and wash fractions were retained for analysis. Purified protein was eluted and concentrated using an ultrafiltration tube (88513, Thermo Fisher Scientific, MA, USA).

### Silver staining

Following electrophoresis, gels were silver-stained (Fast Silver Stain Kit, P0017S, Beyotime, Shanghai, China) using the manufacturer’s protocol. After fixation (50% ethanol/10% acetic acid/40% Milli-Q water), gels were incubated with silver solution and development was terminated once protein bands became visible.

### Synthesis of biotin-labeled cisplatin, biotin pull-down assay and mass spectrometry (MS)

Cisplatin was oxidized by H_2_O_2_ and mixed with biotin in dimethyl sulfoxide (DMSO) by sufficient dissolution and stirred for at least 1 h. 1-Ethyl-3-(3-dimethylaminopropyl) carbodiimide (EDC) and N-hydroxysuccinimide (NHS) were then added to the reaction system and stirred vigorously overnight at room temperature (RT). The final product was washed with an ice-water mixture and finally characterized using ^1^H nuclear magnetic resonance (NMR) spectroscopy. The identification of biotin-labeled cisplatin was shown in Fig. S6A. Breast cancer cells were sufficiently lysed by Lysis buffer and then spun with Streptavidin (SAV) MagBeads (47503ES08, Yeasen-Bio, Shanghai, China) attached with biotin or biotin-labelled Cisplatin for 2 h at 4 °C, and finally the enriched proteins were denatured and used for Western blot analysis or sent to Beijing Institute of Genomics (BGI, Beijing, China) for mass spectrometry.

### RNA immunoprecipitation (RIP) assay

RIP assay was performed according to the protocol provided with the Magna RIP™ RNA-binding protein immunoprecipitation kit (17-700, Millipore, MA, USA). At least 20 million cells were lysed in polysome lysis buffer and subsequently incubated with Anti-IGF2BP2 linked Protein A/G Magnetic Beads for adequate immunoprecipitation. Final RNA was purified and subjected to qRT-PCR analysis.

### RNA stability assay

Total RNA was collected for qRT-PCR after adding actinomycin D (HY-17559, MCE, NJ, USA) at a final concentration of 5 μg/ml according to the time points, and the calculation of RNA half-life was carried out in accordance with the previously reported studies [44].

### Animal ethics statement and subcutaneous tumor xenograft therapy models

Animal experiments were conducted under the supervision and guidance of Institutional Animal Care and Use Committee of Nanjing Medical University, the animal ethical review number of this study was IACUC-2401019. Female BALB/c nude mice (4–6 weeks old, 18–22 g) were randomly grouped (*n* = 5 per group) and each mouse was inoculated with ~1*10^6^ MDA-MB-231 cells in the left mammary fat pad. Ten days later, seven injections of drugs or blank solvents were started according to the experimental protocol: cisplatin (HY-17394, MCE, NJ, USA); WP1130 (HY-13264, MCE, NJ, USA); Corn Oil (HY-Y1888, MCE, NJ, USA); Saline (G4702, Servicebio, China), and tumor size was measured every three days and the volume was calculated according to 1/6*π*(Length)*(Width)^2. We confirm that all experimental procedures and protocols were performed in accordance with the relevant guidelines and regulations, including the ARRIVE guidelines 2.0 and the National Institutes of Health Guide for the Care and Use of Laboratory Animals.

### CCK8 and colony formation assay

The effect of drug combination was tested according to the CCK8 and colony formation assay. For CCK-8 (A311, Vazyme, Nanjing, China), 2500 breast cancer cells/well were seeded in 96-well plates. After 24 h, drug-treated cells received 100 μl of CCK-8 reagent: serum-free DMEM (1:9), incubated for 2 h (light-protected) before measurement. For colony formation, 500 cells/well were plated in 6-well plates, drug-supplemented complete DMEM was added at day 3. After 2 weeks, colonies were stained and counted using crystal violet (C0121, Beyotime, Shanghai, China).

### Assessment of IGF2BP2 activity and association with USP9X protein expression

Based on previously reported IGF2BP2-regulated genes involved in transcript stability, we constructed a gene signature indicative of IGF2BP2 activity, the details can be found in Supplementary Table 5. RNA-seq data (log2 FPKM) from the FUSCC TNBC cohort (https://www.biosino.org/node/project/detail/OEP000155) [30] were used to calculate IGF2BP2 activity scores for each sample using Gene Set Variation Analysis (GSVA). Among these samples, those with available quantitative proteomic data were selected. Based on the median protein expression level of USP9X, samples were stratified into USP9X-low and USP9X-high groups. Differences in IGF2BP2 activity scores between the two groups were assessed using an unpaired two-tailed Student’s *t*-test.

### Public databases and statistical analysis

DUB gene lists were acquired from the Epithelial Systems Biology Laboratory database (https://esbl.nhlbi.nih.gov/Databases/KSBP2/Targets/Lists/DUBs/). TNBC DUB expression profiles were analyzed using bcGenExMiner (http://bcgenex.ico.unicancer.fr). Ubiquitination modification sites were predicted using PhosphoSitePlus (https://www.phosphosite.org) and GPS-Uber (https://gpsuber.biocuckoo.cn/).

Protein intensity data were derived from the Pan-Cancer Proteome Atlas (TPCPA) [45]. For comparisons among breast cancer subtypes, log-transformed mRNA levels were analyzed using the Wilcoxon rank-sum test. Differences in protein detection rates across subtypes were assessed using Fisher’s exact test. To examine the correlation between IGF2BP2 and USP9X in TNBC, Pearson correlation coefficients were calculated using standardized (z-scored) mRNA and protein expression levels. Prior to correlation analysis, missing protein values were imputed using the knn() function from the impute R package (1.82.0).

Survival data were retrieved from the Kaplan–Meier Plotter database (http://kmplot.com/analysis/). Samples were selected according to the following criteria: no prior treatment, ER-negative, PR-negative, HER2-negative, and classified as PAM50 basal-like subtype. Within each group, patients were further stratified based on the optimal cutoff value automatically. Kaplan–Meier survival curves were constructed, and log-rank tests were conducted to assess statistical significance, both implemented using the survival (v3.8-3) and survminer (v0.5.0) packages in R.

Statistical analyses were performed using SPSS 24.0 (IL, USA), GraphPad Prism 9.5 (SD, USA). Two-group comparisons used two-tailed Student’s *t*-tests; multiple comparisons used ANOVA. A *p*-value < 0.05 was considered statistically significant. All experiments were independently repeated ≥3 times unless otherwise stated.

## Supplementary information

Supplementary Figures

Supplementary Tables

Full length uncropped original western blots

## Data Availability

Data supporting the results of this study are available from the corresponding author upon reasonable request. Full length uncropped original Western blots can be found in the Supplementary Material.
