# Supplementary material for: The deubiquitinase USP9X and E3 ligase WWP1 orchestrate IGF2BP2 ubiquitination homeostasis to drive TNBC progression and cisplatin sensitivity
Source: Cell Death Dis. 2025 Oct 6;16(1):703. doi: 10.1038/s41419-025-08038-5 (PMC12500958; doi:10.1038/s41419-025-08038-5)
Supplement: Supplementary file 1 — Supplementary Figures [file 41419_2025_8038_MOESM1_ESM.docx]

**Supplementary Figures**

**The deubiquitinase USP9X and E3 ligase WWP1 orchestrate IGF2BP2 ubiquitination homeostasis to drive TNBC progression and cisplatin sensitivity**

**Figure S1**


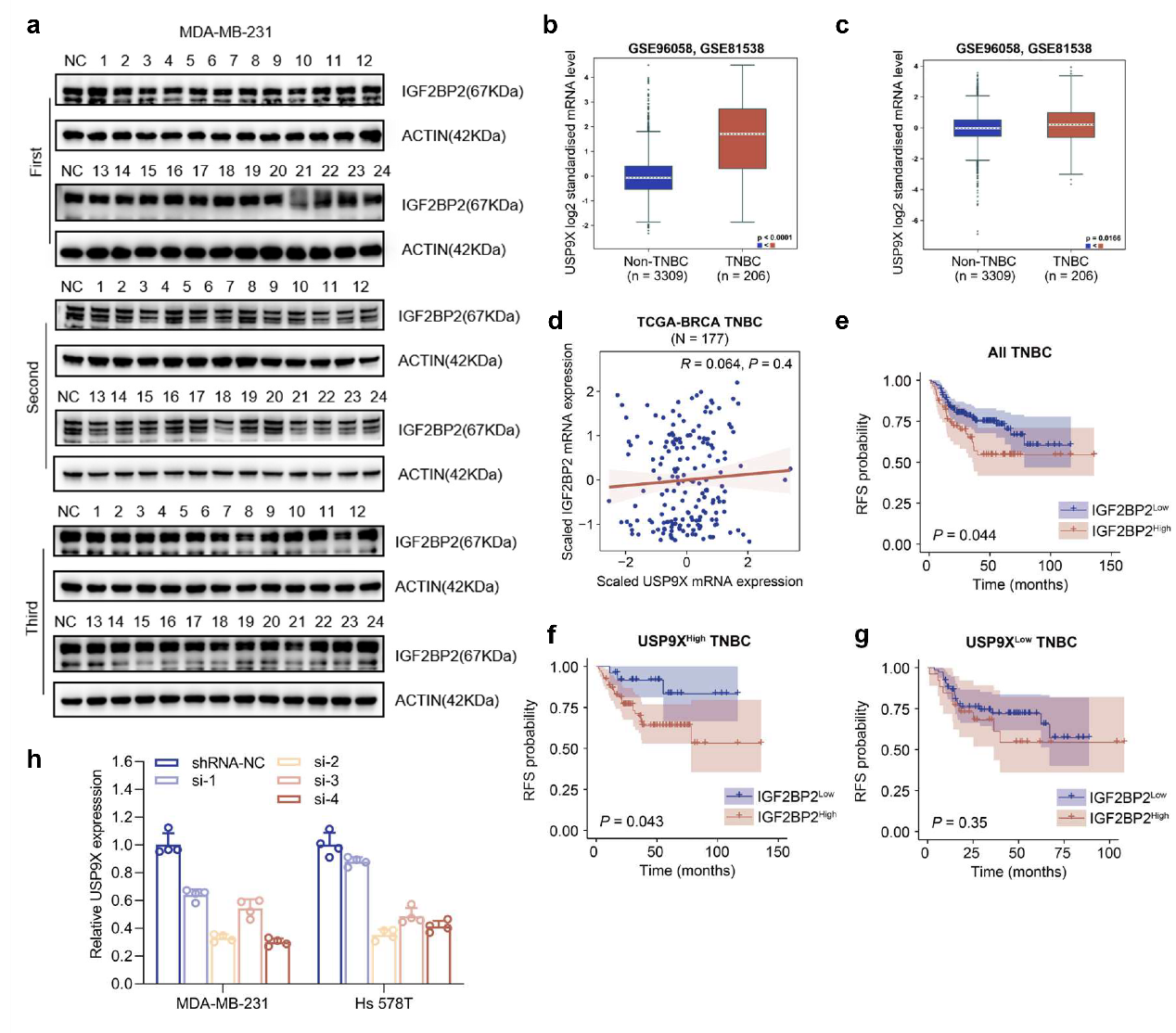


a. After transfection of MDA-MB-231 cells with siDUBs for 72 h, total proteins were collected and analyzed by Western blotting in three independent experiments.

b and c. IGF2BP2 and USP9X mRNA levels were significantly higher in TNBC compared to non-TNBC in GSE96058, GSE81538.

d. No significant correlation was observed between IGF2BP2 and USP9X mRNA levels in TCGA-BRCA TNBC.

e, f and g. Patients were first stratified into three cohorts: the full TNBC cohort, the USP9X^High^ subgroup (expression above the median), and the USP9X^Low^ subgroup (expression below the median). In the full cohort, high IGF2BP2 expression was significantly associated with worse RFS (P = 0.044). This association remained significant in the USP9X^High^ subgroup (P = 0.043), but was not observed in the USP9X^Low^ subgroup (P = 0.35).

h. qRT-PCR analysis of si-USP9X knockdown efficiency.

**Figure S2**


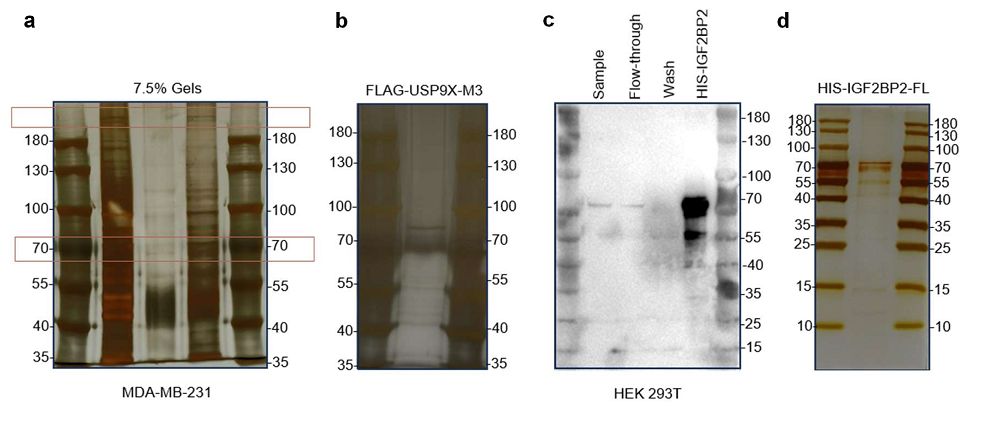


a. MDA-MB-231 cell lysates were pulled down using IgG and IGF2BP2 antibodies and detected by gel electrophoresis using a silver staining kit.

b. Silver staining test of purified GST-USP9X-M3 protein.

c. Western blot was performed on samples from each critical step in the purification of HIS-tagged IGF2BP2 protein to ensure the validity of protein purification.

d. Silver staining test of purified HIS-IGF2BP2 protein.

**Figure S3**


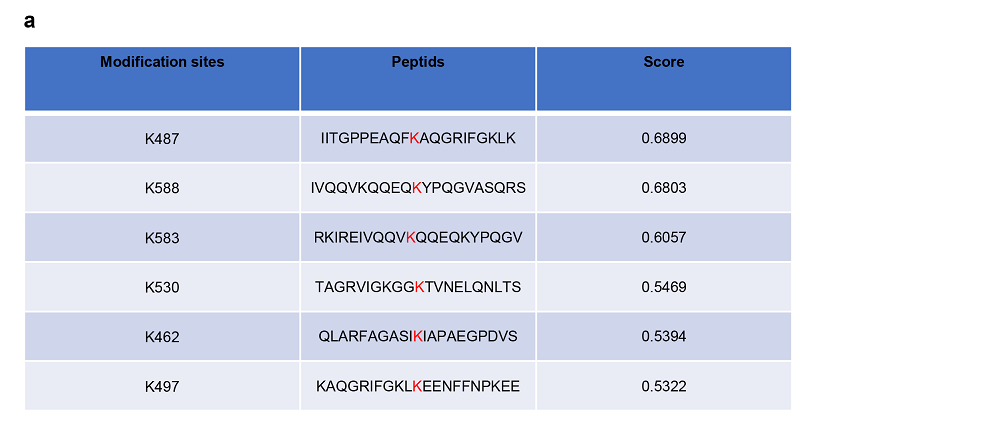


1. Prediction of IGF2BP2 universal and E3-specific lysine ubiquitination sites based on GPS-Uber and PhosphoSitePlus.

**Figure S4**


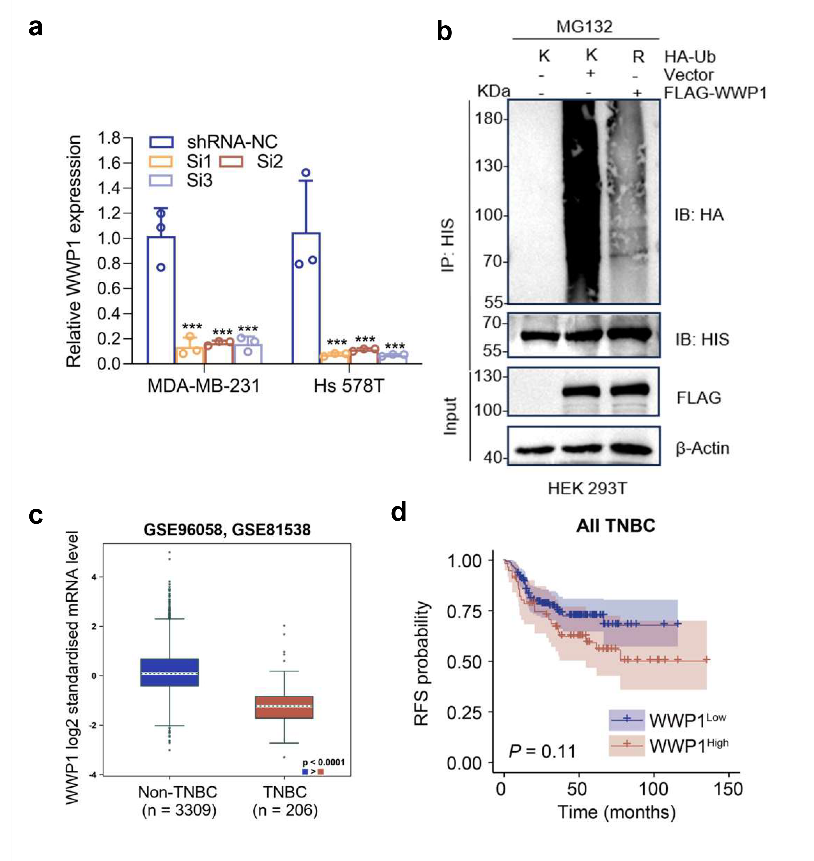


a. qRT-PCR analysis of SiWWP1 knockdown efficiency. Error bars indicate mean (n=3) ± standard deviation. *P<0.05, **P<0.01, ***P<0.001.

b. HIS-tagged IGF2BP2 wild-type or HIS-IGF2BP2 487/583R was used for co-transfection with HA-Ub and FLAG-WWP1, and ubiquitin linkage of IGF2BP2 was analyzed after collecting total proteins after treatment with 20 μM MG-132 for 6 h and after IP pull-down using HIS antibody.

c. WWP1 mRNA expression was significantly downregulated in TNBC compared to non-TNBC samples in both GSE96058 and GSE81538 datasets.

d. Kaplan-Meier analysis of the TNBC cohort revealed no significant association between WWP1 expression levels and patient survival (P=0.11).

**Figure S5**


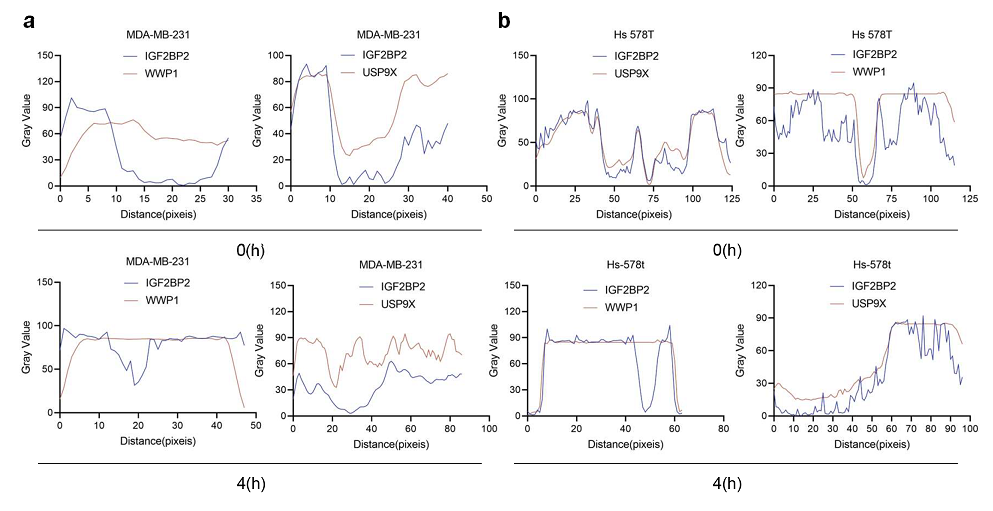


(a and b). Quantitative analysis of co-localized fluorescence intensity of USP9X or WWP1) and IGF2BP2 in MDA-MB-231 and Hs 578T cells after treatment with saline or CDDP for 4h.

**Figure S6**


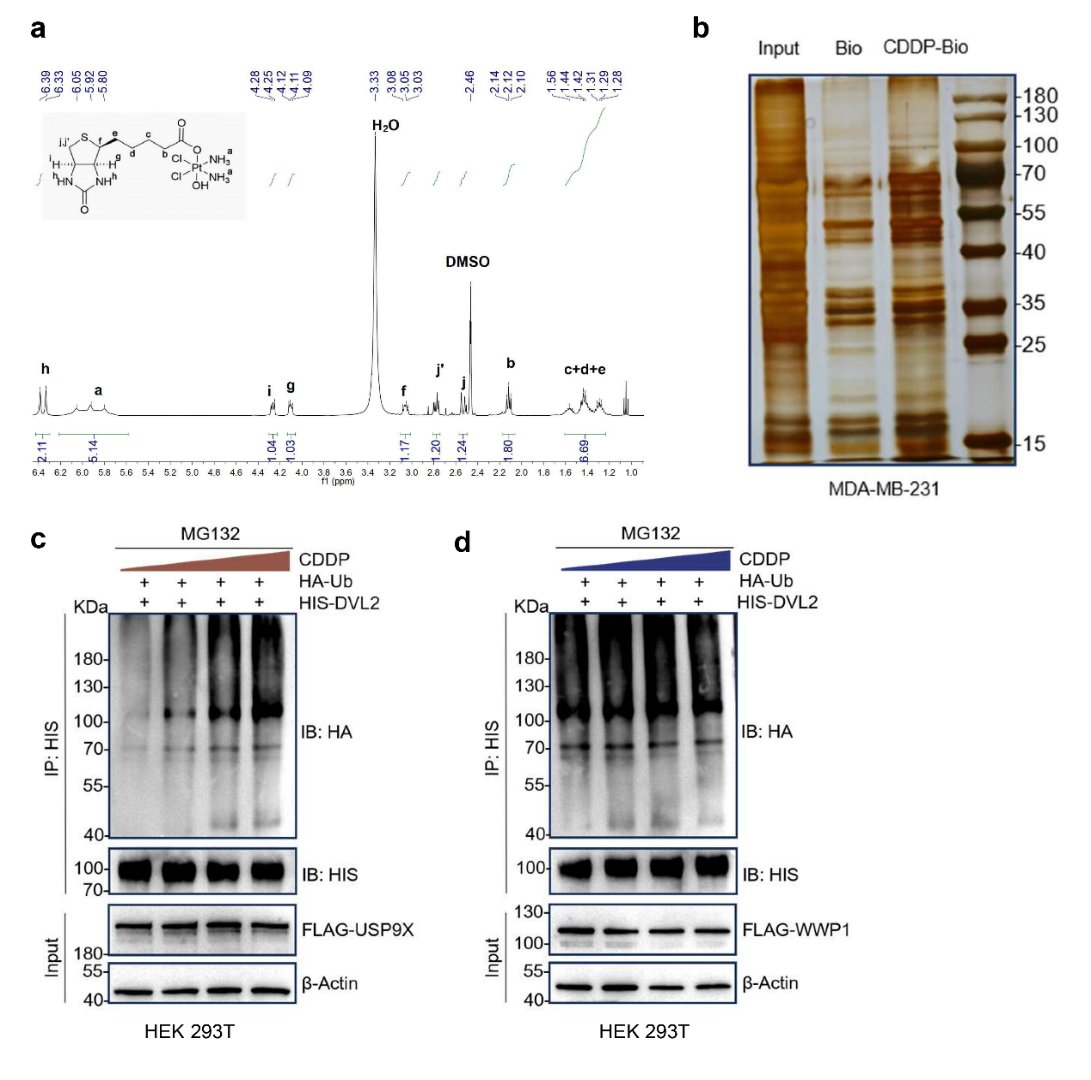


a. ¹H nuclear magnetic resonance (NMR) spectroscopy identification profiles of biotin-labeled cisplatin probes.

b. Silver-staining assay of biotin-labeled cisplatin probes for pull-down experiments.

c and d. HEK 293T cells were co-transfected with His-tagged DVL2, HA-ubiquitin (HA-Ub), and either FLAG-tagged USP9X or FLAG-tagged WWP1. Following treatment with CDDP(0-100 μM gradient) and 20 μM MG-132, total proteins were harvested. His-tagged proteins were immunoprecipitated using anti-His antibody, and DVL2 ubiquitination was detected by immunoblotting with anti-HA antibody.

**Figure S7**


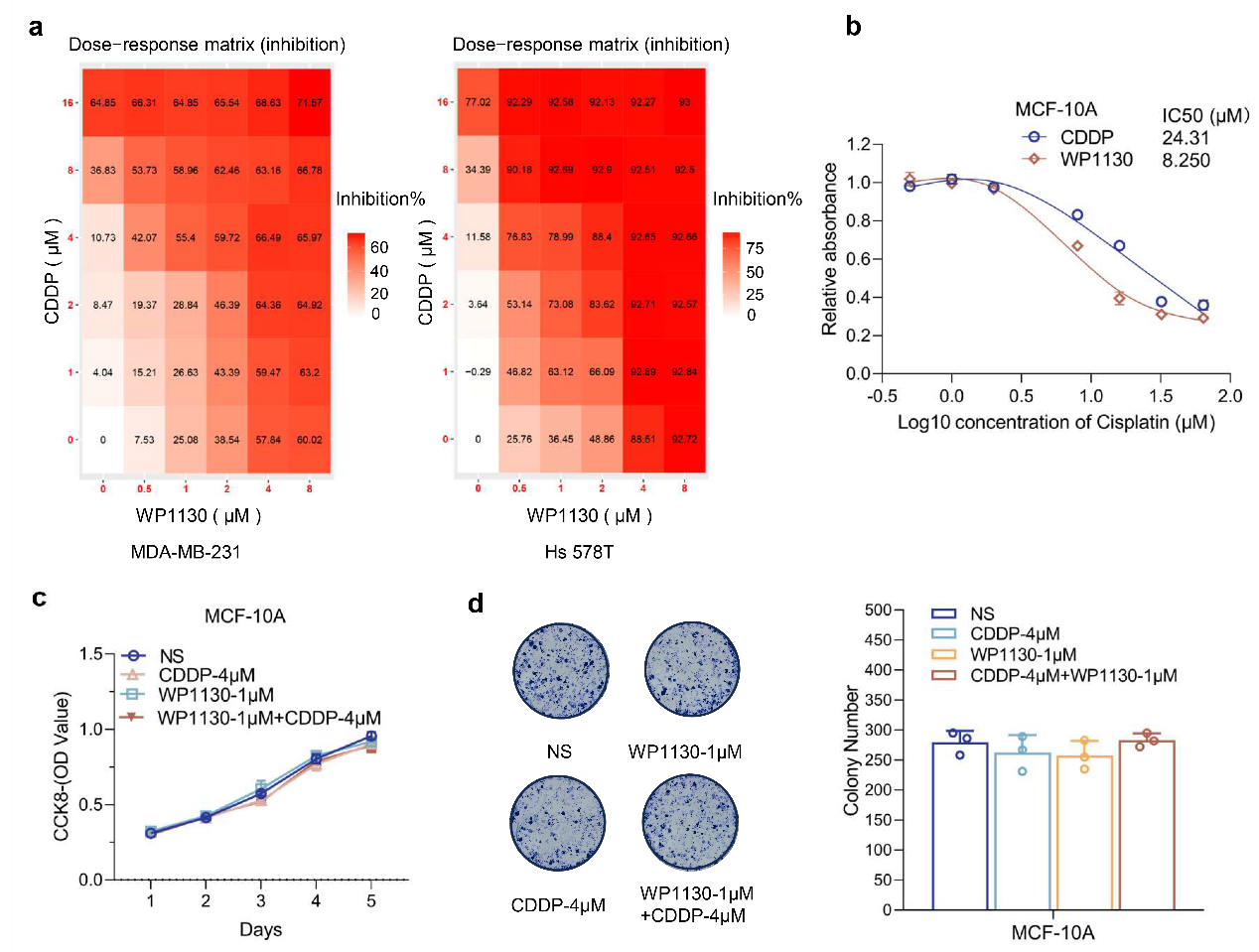


1. Inhibition matrix of drug combination of CDDP and WP1130.
2. The CCK-8 assay was used to determine the IC50 values of Cisplatin and WP1130 in MCF-10A cells.
3. Growth of MCF-10A cells after 5 days of treatment with different drugs was measured using the CCK8 assay.
4. Cell growth was measured using a colony formation assay over 14 days of different drug treatments and statistically analyzed after counting.
