# Supplementary material for: The deubiquitinase USP9X and E3 ligase WWP1 orchestrate IGF2BP2 ubiquitination homeostasis to drive TNBC progression and cisplatin sensitivity
Source: Cell Death Dis. 2025 Oct 6;16(1):703. doi: 10.1038/s41419-025-08038-5 (PMC12500958; doi:10.1038/s41419-025-08038-5)
Supplement: Supplementary file 2 — Supplementary Tables [file 41419_2025_8038_MOESM2_ESM.docx]

**Supplementary Table****s**

**The deubiquitinase USP9X and E3 ligase WWP1 orchestrate IGF2BP2 ubiquitination homeostasis to drive TNBC progression and cisplatin sensitivity**

1. **DUBs expression in TNBC**

| **NAME** | **Non-TNBC Mean** | **TNBC Mean** | **P.Value** | **Status** | **Rename** |
| --- | --- | --- | --- | --- | --- |
| OTULIN | -3.00E-04 | 1.1962 | ＜0.001 | TNBC High | 1 |
| USP6NL | -8.10E-02 | 1.1867 | ＜0.001 | TNBC High | 2 |
| USP1 | -1.04E-02 | 1.2389 | ＜0.001 | TNBC High | 3 |
| USP49 | -5.91E-02 | 0.9502 | ＜0.001 | TNBC High | 4 |
| USP39 | 1.02E-02 | 0.8743 | ＜0.001 | TNBC High | 5 |
| USP18 | 1.19E-01 | 0.6836 | ＜0.001 | TNBC High | 6 |
| USP28 | -1.06E-01 | 0.502 | ＜0.001 | TNBC High | 7 |
| USP10 | 1.12E-01 | 0.6865 | ＜0.001 | TNBC High | 8 |
| USP31 | -4.20E-02 | 0.5792 | ＜0.001 | TNBC High | 9 |
| OTUD6B | 6.92E-02 | 0.6323 | ＜0.001 | TNBC High | 10 |
| USP43 | -4.40E-02 | 0.4522 | ＜0.001 | TNBC High | 11 |
| USP15 | -7.90E-03 | 0.4046 | ＜0.001 | TNBC High | 12 |
| USP2 | -2.57E-02 | 0.3373 | ＜0.001 | TNBC High | 13 |
| USP24 | -4.75E-02 | 0.304 | ＜0.001 | TNBC High | 14 |
| USP13 | 5.77E-02 | 0.4517 | ＜0.001 | TNBC High | 15 |
| USP44 | 1.17E-01 | 0.5427 | ＜0.001 | TNBC High | 16 |
| USP54 | -4.50E-03 | 0.2285 | 0.0125 | TNBC High | 17 |
| OTUD4 | -4.91E-02 | 0.3072 | 0.0003 | TNBC High | 18 |
| USP36 | 4.47E-02 | 0.2687 | 0.0117 | TNBC High | 19 |
| USP5 | 2.14E-02 | 0.2721 | 0.0206 | TNBC High | 20 |
| USP9X | -4.19E-02 | 0.1772 | 0.0166 | TNBC High | 21 |
| USP25 | -9.44E-02 | 0.2099 | 0.0002 | TNBC High | 22 |
| USP34 | -8.94E-02 | 0.1423 | 0.0123 | TNBC High | 23 |
| USP45 | -8.94E-02 | 0.1423 | 0.0104 | TNBC High | 24 |
| USP42 | -0.0077 | 0.1423 | 0.0871 | Relative Same |  |
| USP17L8 | 0.0822 | 0.2427 | 0.2559 | Relative Same |  |
| USP29 | 5.14E-01 | 5.61E-01 | 0.5169 | Relative Same |  |
| USP17L15 | 2.21E-01 | 2.93E-01 | 0.4601 | Relative Same |  |
| USP26 | 5.94E-01 | 6.33E-01 | 0.6142 | Relative Same |  |
| USP27X | 5.10E-03 | 1.22E-01 | 0.1646 | Relative Same |  |
| USP41 | NA | NA | NA | FLASE |  |
| USP17L4 | 6.72E-02 | 6.53E-02 | 0.9767 | Relative Same |  |
| USP17L20 | 1.93E-02 | 0.00E+00 | 0.3174 | Relative Same |  |
| USP17L17 | 1.93E-02 | 0.00E+00 | 0.3174 | Relative Same |  |
| USP17L13 | 2.33E-02 | 0.00E+00 | 0.2474 | Relative Same |  |
| USP17L3 | 9.72E-02 | 7.06E-02 | 0.7157 | Relative Same |  |
| USPL1 | -4.56E-02 | -4.10E-02 | 0.9543 | Relative Same |  |
| USP17L22 | 5.61E-02 | 0.00E+00 | 0.0028 | Relative Same |  |
| USP17L10 | 1.33E-01 | 0.073 | 0.2813 | Relative Same |  |
| OTUD3 | -7.32E-02 | -1.27E-01 | 0.5922 | Relative Same |  |
| USP17L7 | 4.37E-01 | 3.53E-01 | 2.60E-01 | Relative Same |  |
| OTUD6A | 1.61E-01 | 8.65E-02 | 0.2844 | Relative Same |  |
| USP48 | -8.69E-02 | -8.48E-02 | 0.9803 | Relative Same |  |
| USP11 | -1.34E-02 | -1.09E-01 | 0.3327 | Relative Same |  |
| USP21 | 5.74E-02 | -3.20E-03 | 0.4518 | Relative Same |  |
| USP16 | -1.00E-03 | -1.04E-01 | 0.1881 | Relative Same |  |
| OTUB1 | 3.27E-02 | -1.46E-01 | 0.0182 | TNBC Low |  |
| USP9Y | 9.28E-02 | 0.0167 | ＜0.001 | TNBC Low |  |
| USP37 | -1.96E-02 | -2.20E-01 | 0.0321 | TNBC Low |  |
| USP14 | 1.70E-01 | -0.0326 | 0.0027 | TNBC Low |  |
| OTUD5 | 1.44E-02 | -2.51E-01 | 0.0031 | TNBC Low |  |
| USP4 | -1.05E-01 | -4.31E-01 | ＜0.001 | TNBC Low |  |
| USP50 | -5.25E-02 | -3.76E-01 | ＜0.001 | TNBC Low |  |
| OTUB2 | 5.23E-02 | -3.30E-01 | ＜0.001 | TNBC Low |  |
| USP3 | 1.40E-03 | -4.12E-01 | ＜0.001 | TNBC Low |  |
| USP33 | 4.89E-02 | -2.15E-01 | 2.00E-04 | TNBC Low |  |
| USP22 | 4.20E-03 | -4.15E-01 | ＜0.001 | TNBC Low |  |
| USP12 | -2.00E-03 | -4.09E-01 | ＜0.001 | TNBC Low |  |
| USP51 | -8.60E-03 | -4.76E-01 | ＜0.001 | TNBC Low |  |
| USP32 | 2.00E-01 | -3.03E-01 | ＜0.001 | TNBC Low |  |
| OTUD7A | 4.39E-02 | -4.25E-01 | ＜0.001 | TNBC Low |  |
| USP53 | 4.69E-02 | -4.82E-01 | ＜0.001 | TNBC Low |  |
| USP20 | -1.96E-02 | 5.87E-01 | ＜0.001 | TNBC Low |  |
| USP46 | -1.82E-02 | -6.64E-01 | ＜0.001 | TNBC Low |  |
| OTUD1 | 4.77E-02 | -5.01E-01 | ＜0.001 | TNBC Low |  |
| USP40 | -1.84E-02 | -7.37E-01 | ＜0.001 | TNBC Low |  |
| USP19 | -9.50E-02 | -7.16E-01 | ＜0.001 | TNBC Low |  |
| USP35 | 2.38E-01 | -4.26E-01 | ＜0.001 | TNBC Low |  |
| USP38 | 5.67E-02 | -5.96E-01 | ＜0.001 | TNBC Low |  |
| USP47 | -3.45E-02 | -7.05E-01 | ＜0.001 | TNBC Low |  |
| USP7 | 1.22E-01 | -5.11E-01 | ＜0.001 | TNBC Low |  |
| USP8 | -9.00E-04 | -8.83E-01 | ＜0.001 | TNBC Low |  |
| OTUD7B | 8.09E-02 | -0.8334 | ＜0.001 | TNBC Low |  |
| USP30 | 2.21E-02 | -1.0657 | ＜0.001 | TNBC Low |  |

1. **Sequence of siRNAs and shRNAs**

| **Sequence of siWWP1** | | | |
| --- | --- | --- | --- |
| **Name** | **Rename** | **Sense** | **Antisense** |
| hWWP1-600 | Si1 | GGUUCGGAACAGCAAUAUA | UAUAUUGCUGUUCCGAACC |
| hWWP1-2031 | Si2 | GGUACUUUGUUGAUCAUAA | UUAUGAUCAACAAAGUACC |
| hWWP1-1790 | Si3 | GGAGCUAUGCAACAGUUUA | UAAACUGUUGCAUAGCUCC |
| **Sequence of siUSP9X** | | | |
| **Name** | **Rename** | **Sense** | **Antisense** |
| USP9X-Homo-1190 | Si1 | GGGCUAACAAUAUCAUUCATT | UGAAUGAUAUUGUUAGCCCTT |
| USP9X-Homo-1395 | Si2 | GUCCAUGUGAAUCAGUUUCTT | GAAACUGAUUCACAUGGACTT |
| USP9X-Homo-2101 | Si3 | GACCUUACAGGAUCUUGAUTT | AUCAAGAUCCUGUAAGGUCTT |
| USP9X-Homo-2517 | Si4 | GGGUUAUUCCCGCACUGAATT | UUCAGUGCGGGAAUAACCCTT |

1. **Primer sequence**

| **Gene Name** | **Forward** | **Reverse** |
| --- | --- | --- |
| USP9X | GTCGGAGGGAATGACAACC | AAACTGGAACCACCCATCG |
| IGF2BP2 | CTACGCCTTCGTGGACTACC | CATCCAACACCTCCCACTG |
| β-Actin | TCACCCACACTGTGCCCATCTACGA | CAGCGGAACCGCTCATTGCCAATGG |
| WWP1 | GCTGTTGAAGGCACGAATG | AGGCTCAGATGCGAGTGGT |
| MYC | TGCTCCATGAGGAGACACC | CTTTTCCACAGAAACAACATCG |
| CDK6 | CGTGGTCAGGTTGTTTGATG | CCTCGGAGAAGCTGAAACAT |

1. **Antibodies**

| **Antibodies** | **Identifier** | **Source** | **Location** |
| --- | --- | --- | --- |
| IGF2BP2 | ab128175 | Abcam | Cambridge, UK |
| USP9X | 55054-1-AP | Protein-tech | Wuhan, China |
| β-Actin | 66009-1-Ig | Protein-tech | Wuhan, China |
| 6*His  (HIS) | 66005-1-Ig/10001-0-AP | Protein-tech | Wuhan, China |
| DYKDDDDK tag Polyclonal antibody (Binds to FLAG® tag epitope) | 20543-1-AP/ 66008-4-Ig | Protein-tech | Wuhan, China |
| HA | 51064-2-AP | Protein-tech | Wuhan, China |
| WWP1 | 28689-1-AP | Protein-tech | Wuhan, China |
| CDK6 | 13331S | Cell Signaling Technology | MA, USA |
| EIF4A1 | ab31217 | Abcam | Cambridge, UK |
| HUR | 11910-1-AP | Protein-tech | Wuhan, China |

1. **IGF2BP2-related genes**

| **GENE** | **TITLE** | **PUBMED ID** |
| --- | --- | --- |
| FOXM1 | m6A modification of lncRNA ABHD11-AS1 promotes colorectal cancer progression and inhibits ferroptosis through TRIM21/IGF2BP2/ FOXM1 positive feedback loop | 38838765 |
| FSCN1 | Recognition of RNA N6-methyladenosine by IGF2BP proteins enhances mRNA stability and translation | 29476152 |
| GPT2 | The m6A reader IGF2BP2 regulates glutamine metabolism and represents a therapeutic target in acute myeloid leukemia | 36306790 |
| HMGA1 | LINC00460/DHX9/IGF2BP2 complex promotes colorectal cancer proliferation and metastasis by mediating HMGA1 mRNA stability depending on m6A modification | 33526059 |
| LDHA | EIF4A3-Induced circARHGAP29 Promotes Aerobic Glycolysis in Docetaxel-Resistant Prostate Cancer through IGF2BP2/c-Myc/LDHA Signaling | 34965937 |
| MYC | Recognition of RNA N6-methyladenosine by IGF2BP proteins enhances mRNA stability and translation | 29476152 |
| SLC1A5 | The m6A reader IGF2BP2 regulates glutamine metabolism and represents a therapeutic target in acute myeloid leukemia | 36306790 |
| SNAI1 | FTO Inhibits Epithelial Ovarian Cancer Progression by Destabilising SNAI1 mRNA through IGF2BP2 | 36358640 |
| TAB3 | Inhibition of METTL3 attenuates renal injury and inflammation by alleviating TAB3 m6A modifications via IGF2BP2-dependent mechanisms | 35417191 |
| TK1 | Recognition of RNA N6-methyladenosine by IGF2BP proteins enhances mRNA stability and translation | 29476152 |
