# Supplementary figures and images for: The deubiquitinase USP9X and E3 ligase WWP1 orchestrate IGF2BP2 ubiquitination homeostasis to drive TNBC progression and cisplatin sensitivity
Source: Cell Death Dis. 2025 Oct 6;16(1):703. doi: 10.1038/s41419-025-08038-5 (PMC12500958; doi:10.1038/s41419-025-08038-5)

**Figure 1**


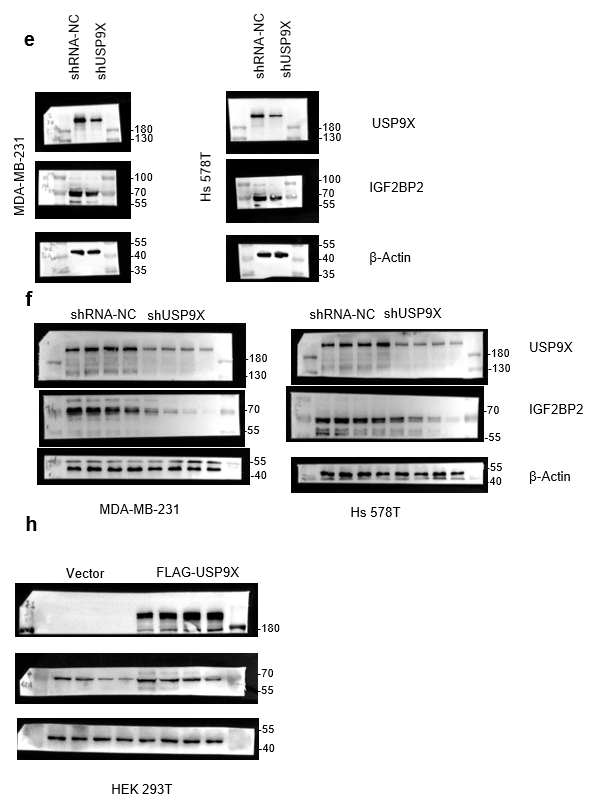


**Figure 2**


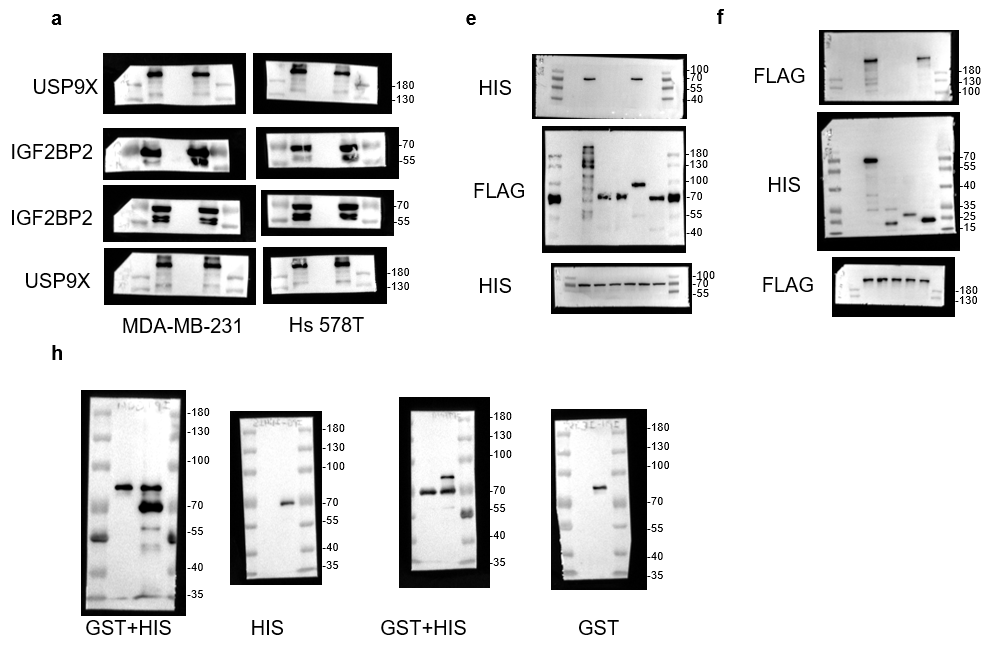


**Figure 3**


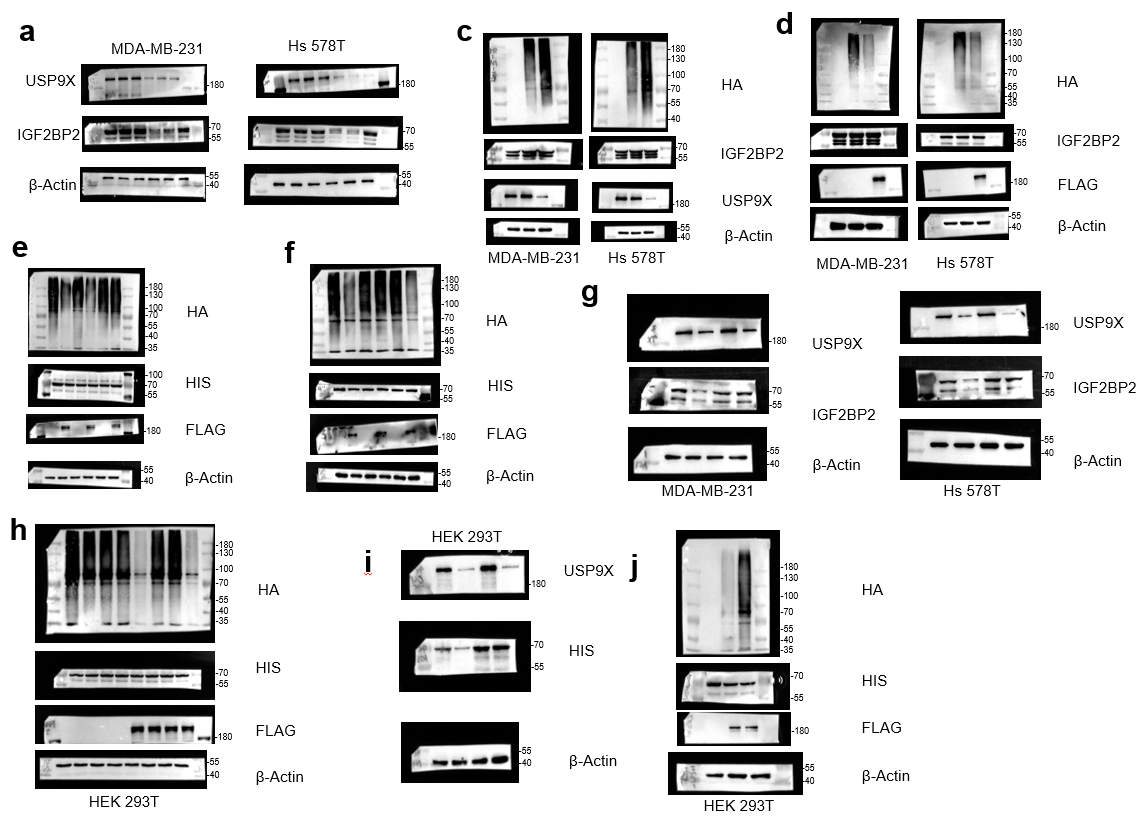


**Figure 4**


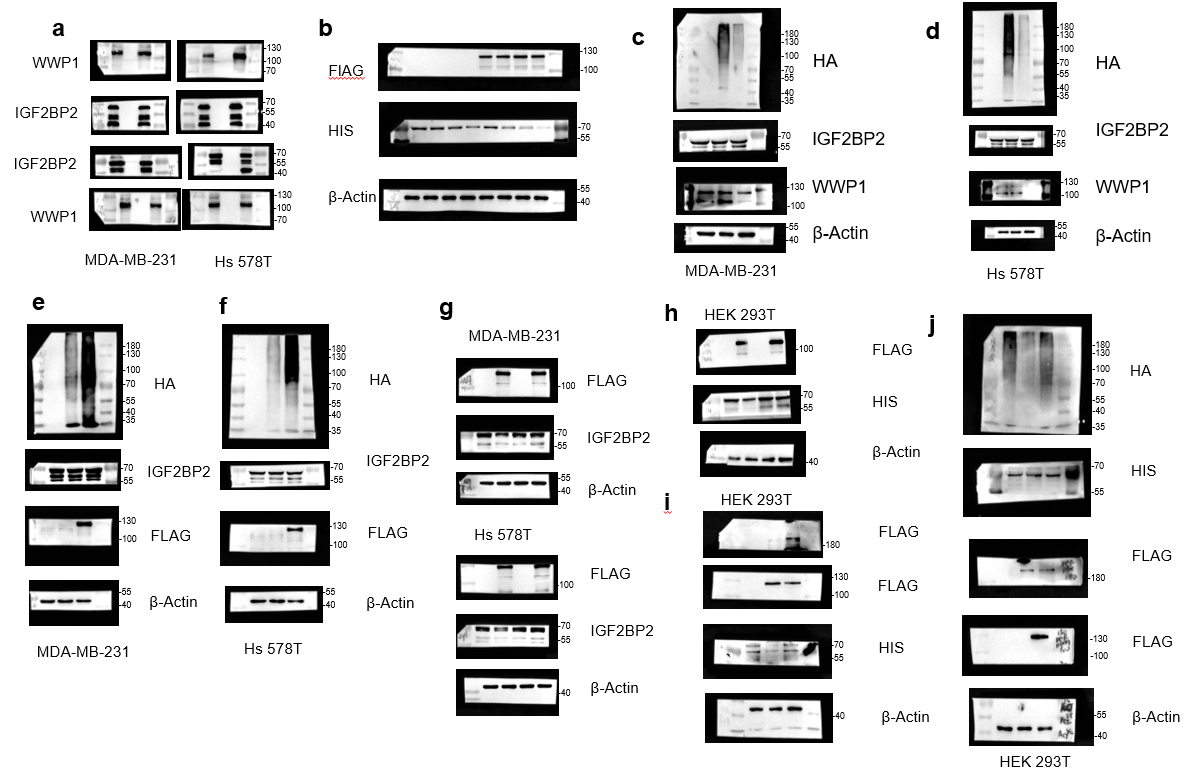


**Figure 5**


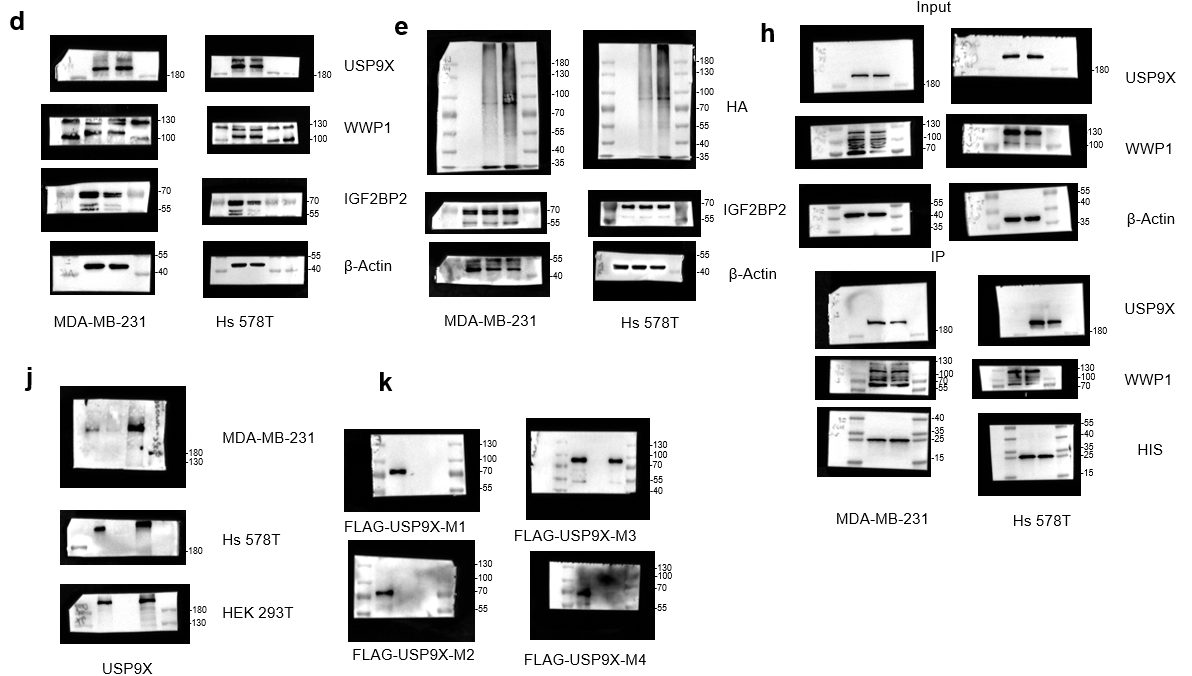


**Figure 6**


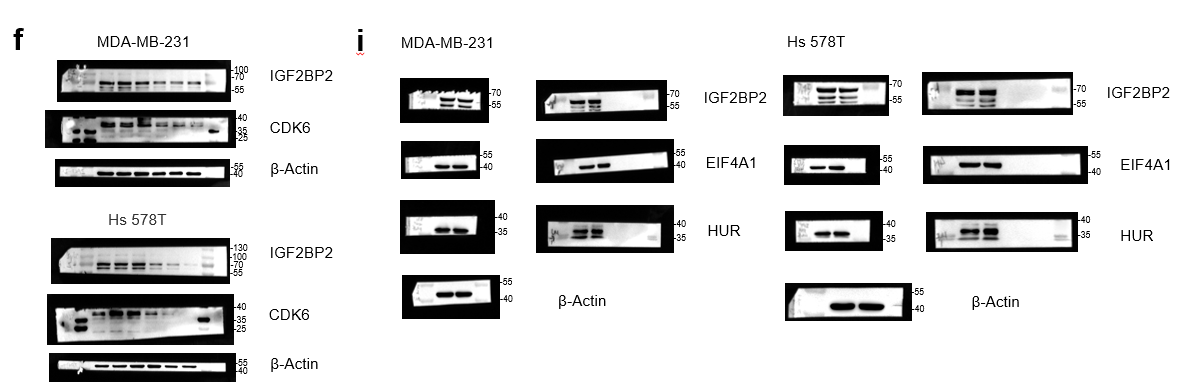


**Figure S1**

**
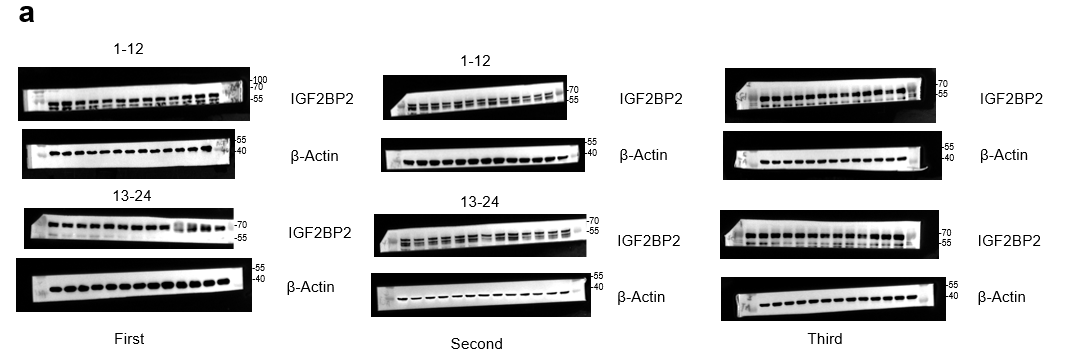
**

**Figure S2**

**
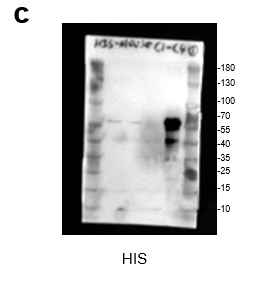
**

**Figure S4**

**
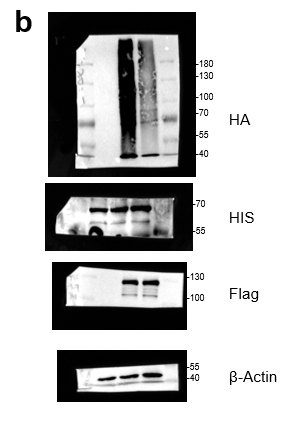
**

**Figure S6**

**
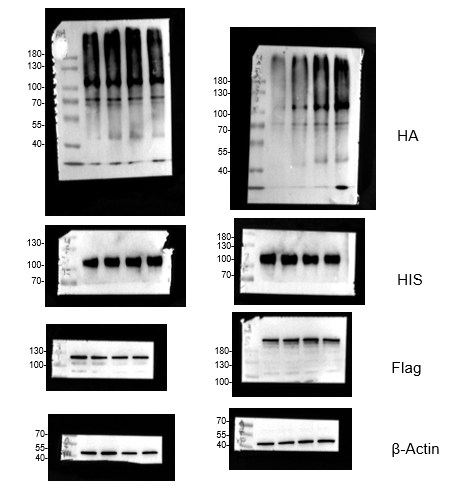
**

Supplement: Supplementary file 3 — Full length uncropped original western blots [file 41419_2025_8038_MOESM3_ESM.docx]
